# Clinical utility of GAL-8, ITGΒ-1, and HIF-1α as non-invasive diagnostic and prognostic biomarkers for assessing glioma

**DOI:** 10.1007/s11060-025-05110-0

**Published:** 2025-06-13

**Authors:** Taylan Turan, Ö. H. Emmez, A. M. Kaymaz, A. Gönenç

**Affiliations:** 1 Department of Biochemistry, Faculty of Pharmacy, Gazi University, Ankara, Turkey; 2 Department of Neurosurgery, Faculty of Medicine, Gazi University, Ankara, Turkey

**Keywords:** Glioma, GAL-1, -3, -8, ITGβ-1, HIF-1α, MMP-2 and − 9

## Abstract

**Purpose:**

Gliomas have attracted attention as the most common primary tumor of the CNS. Gliomas are made up of glial components of the nervous system. They cause more than 40% of CNS neoplasms, with an increased incidence in the aged 65 years and older. Despite advances in the characterization of the pathogenesis of these tumors, gliomas remain incurable. In recent years, scientific developments have shifted the attention of scientists to the potentials of galectins in glioma biology. Many studies have reported the important functions of galectins in cancer biology, apoptosis, and escape from tumor immunity, metastasis, and angiogenesis. Although few studies in the literature evaluate galectin expressions in gliomas, in-vitro and in-vivo, no study evaluating galectin serum levels in the clinic has been reported. Therefore, within the scope of this project, the roles of GAL-1, GAL-3, and GAL-8 in glioma pathogenesis and the possible link between gliomagenesis and the serum levels of ITGβ-1, HIF-1α, MMP-2, and − 9 parameters associated with hypoxia, angiogenesis, and migration, were evaluated in newly diagnosed and untreated LGG and HGG.

**Methods:**

The study included 50 HGG and 50 LGG patients and 50 healthy controls with mean ages of 55.48 ± 1.51, 41.04 ± 1.66 and 39.20 ± 1.20 years, respectively.

**Results:**

Serum GAL-1, -3, -8, ITGβ-1, HIF-1α, MMP-2, and − 9 levels significantly differed between the glioma and healthy control groups. In addition, serum GAL-1, -3, -8, ITGβ-1, MMP-2 and − 9 levels were significantly greater in HGG compared to LGG. Although there was no significant difference in serum HIF-1α levels between the groups with respect to tumor grade, an increase in HGG was observed. This study is the first in the Turkish population in which serum GAL-1, -3, and − 8 levels were clinically evaluated together in glioma pathologies with high angiogenic activity and sheds light on the role of increased serum galectin levels in the promotion of low-grade tumors to high-grade tumors.

**Conclusion:**

In this respect, we believe that GAL-8, ITGβ-1, and HIF-1α may be usable as a panel of non-invasive biomarkers for glioma diagnosis. Additionally, this study may contribute to studies on glioma treatment and the inhibition of gliomagenesis by bringing a new perspective on the targeting galectins.

## Background

Gliomas are the most common primary tumors of the Central Nervous System (CNS) [1] and account for more than 50% of all primary brain tumors [2], according to the WHO classification of CNS tumors. The incidence of gliomas increases significantly with age, particularly after 65 years [3].

Galectins are calcium-independent, small molecular weight, soluble lectin family members that bind to carcinoembryogenic antigens and laminins and play a role in cell growth and activation. They are involved in various processes, including cell growth regulation, apoptosis, cell adhesion, embryonic development, and inflammation [4]. Galectin-1 (GAL-1) released from adipose tissue, stromal cells, lymph nodes, and endothelial cells induces activated T-cell apoptosis in a caspase-8 and − 9 dependent manner [5, 6]. Galectin-3 (GAL-3), characterized from the outer membrane of macrophages, has dual effects on apoptosis. It induces apoptosis and metastasis through caspase-9 activation, like GAL-1, and has antiapoptotic properties [7]. Galectin-8 (GAL-8) induces apoptosis in T cells via Fas ligand expression and causes caspase-mediated apoptosis [8]. Certain galectins that contribute directly to cancer progression have emerged as potential targets for the advancement of innovative treatment strategies against cancer.

Integrins function as transmembrane receptors, orchestrating both the physical and biochemical interactions between cells and the surrounding ECM. Galectins and integrins interact closely cooperate in regulating cell adhesion and/or migration. The interaction of galectins with integrin β1 (ITGβ1) appears to play a critical role in tumor progression, apoptosis, angiogenesis, and migration [9, 10] through cell adhesion, cell motility, and intracellular signaling pathways [10]. For this reason, we aimed to reveal the potential of these galectins, which are known to modulate ITGβ-1 expression, as therapeutic targets for each type of glioma.

The process of cell migration entails three independents but interrelated biological mechanisms: cell adhesion to various receptors of the extracellular matrix (ECM), cell motility involving reorganization of the actin cytoskeleton by modification of components of the adhesion complex, and invasion involving degradation of matrix proteins by tumor-secreted proteolytic serine proteases, cathepsins and matrix metalloproteinases (MMPs) [11]. Galectins are involved in all these cell migration stages [12]. GAL-1, -3 and − 8 have been shown to affect glioma cell migration [13].

MMPs constitute a family of structurally related enzymes that are involved in the breakdown of various ECM components, including fibrillar and non-fibrillar collagens, fibronectin, laminin, and basement membrane proteoglycans and at least 20 members have been identified in humans [14]. MMP-2 differs from other MMPs in terms of being expressed by many cells, its widespread tissue distribution, and its activation mode. MMP-2 regulates cell proliferation, adhesion, and migration [15], and its expression has been shown to increase in high-grade tumors. MMP-9, a neutral protease first isolated from human neutrophils, can degrade natural type-I collagen, proteoglycans, and laminins.

Recent studies have shown that necrotic areas are surrounded by pseudopalyzing cells, which are considered poor prognostic features [16, 17], and that these pseudopalyzates are severely hypoxic, overexpress hypoxia-inducible factor-1α (HIF-1α) and secrete proangiogenic factors such as VEGF and IL-8. While HIF-1α is rapidly degraded in proteasomes by ubiquitin incorporation under normoxia conditions, it passes from the cytoplasm to the nucleus under hypoxia conditions, where it induces the transcription of its target genes [18]. HIF-1α, which is closely associated with gliomagenesis, may act as a potent activator of angiogenesis and invasion through excessive transcription of some critical target genes [18, 19].

The most important factor underlying the difficulty of gliomas in surgical treatment is the ability of glioma cells to migrate through narrow extracellular spaces in the brain actively and generally travel relatively long distances. In addition, even after surgical resection and adjuvant treatment of gliomas, tumor cells remaining around the removed tumor area can cause the tumor to recur [20]. For this reason, despite the testing of new adjuvant treatment options in addition to tumor cell resection and developments in characterizing the pathogenesis of gliomas, these tumors remain untreated [21]. Although few studies in the literature evaluating galectin expression levels in gliomas have reported conflicting results in vitro and in vivo [13, 22], no studies evaluating the serum levels of galectins in the clinic have been reported. The aim of this study was to fill this gap in the literature by investigating serum samples collected from the Turkish population. Within the scope of the study, which focused on galectins and their potential effects on glioma, serum galectin levels were measured in newly diagnosed and untreated low-grade gliomas (LGGs) and high-grade gliomas (HGGs). The possible relationship of these proteins with tumor grade was evaluated to determine their potential as target biomolecules not only for diagnosis but also for prognosis. In addition, the serum levels of ITGβ-1, HIF-1α, MMP-2, and − 9, which are parameters related to hypoxia, angiogenesis, and migration, were measured and compared in LGG and HGG.

## Methods

### Study groups

The study included a total of 100 adult patients, comprising 50 patients diagnosed with LGG and 50 with HGG in their supratentorial region. The type and grade of gliomas were confirmed through pathological diagnosis in accordance with the WHO criteria. None of the patients had started radiotherapy/chemotherapy (RT/CT) treatment prior to their inclusion in the study. Additionally, 50 healthy individuals over the age of 18 without any systemic diseases were included as the control group. Approval for the study was obtained from the Gazi University Clinical Research Ethics Committee with the decision numbered 2021-15/558 dated 23.06.2021, and written informed consent was obtained from all participants.

### Collection of blood samples

Blood samples from the individuals constituting the study group were collected in red-capped plain serum tubes and transferred to the research laboratory with a cold chain. Then, these blood samples were centrifuged at 4500 rpm for 15 min. to obtain the serum. Then, the serums were aliquoted separately in Eppendorf tubes and stored in an 80^o^C freezer until serum GAL-1, -3, -8, ITGβ-1, HIF-1α, MMP-2, and − 9 ELISA measurements were performed.

### Enzyme-linked immunosorbent assay (ELISA)

Commercially purchased ELISA kits (Elabscience, USA) were used for serum measurements of GAL-1, -3 and − 8; hypoxia; angiogenesis; and the migration-related parameters ITGβ-1, HIF-1α, MMP-2, and − 9, and measurements were carried out in accordance with the kit procedure.

To determine the serum GAL-1, -3, -8, ITGβ-1, HIF-1α, MMP-2, and − 9 levels of the study groups, 100 µL of standard/control/sample was added to each well; the plate was covered, and incubated in an incubator at 37 °C for 90 min. After incubation, the liquid in the wells was poured, 100 µL Biotinylated Detection Antibody was added to each well, and the plate was covered again and incubated at 37 °C for 60 min. The wells were then washed three times with washing solution. 200 µL of GAL-1, -3, -8, ITGβ-1, HIF-1α, MMP-2, and − 9 conjugates were added to each well and incubated in an incubator at 37 °C for 30 min, after which the wells were washed five times with washing solution. Then 90 µL of substrate solution was added to each well, covered with aluminum foil, and incubated at 37 °C for 15 min. The reaction was terminated by the addition of 50 µL of stop solution. The absorbances were measured at 450 nm on an ELISA reader. A calibration graph corresponding to the concentration values was obtained from the absorbance measurements. The concentrations of samples with unknown absorbances were determined from this calibration graph.

#### GAL-1, -3 and − 8 assays

Human serum GAL-1 levels were measured using a commercial ELISA kit (Elabscience Biotechnology Co. Ltd., USA, E-EL-H1051) following the manufacturer’s protocol. The assay demonstrated a sensitivity of 0.47 ng/mL and a detection range of 0.78 to 50 ng/mL. No significant cross-reactivity or interference was detected in this assay. All measurements were performed in duplicate to ensure accuracy and reliability. The intra-assay coefficient of variation (CV) was less than 4.7%, while the inter-assay precision was less than 6.5%.

Commercial ELISA kits (Elabscience Biotechnology Co. Ltd., USA, E-EL-H1470) were used for the quantification of serum levels of GAL-3. All the tests were performed according to the manufacturer’s instructions. The kit sensitivity for GAL-3 was 0.09 ng/mL, and the detection range was between 0.16 and 10 ng/mL. Cross-reactivity and interference were not detected in the reaction. To ensure accuracy and reliability, all the measurements were conducted in duplicate. The intra-assay CV was < 4.1% and inter-assay precision was < 5.0%.

Serum GAL-8 concentrations were determined with a commercially available ELISA (Elabscience Biotechnology Co. Ltd., USA, E-EL-H14094) according to the manufacturer’s instructions. The sensitivity of the human GAL-8 ELISA kit was 0.10 ng/mL, with a detection range of 0.32-20 ng/mL. No evidence of cross-reactivity or interference was observed. All the determinations were carried out in duplicate to enhance the measurement precision. The intra- and inter-assay CVs were controlled between < 5.7% and < 6.1%, respectively.

#### ITGβ-1 assay

Using a sandwich-ELISA (Elabscience Biotechnology Co. Ltd., USA, E-EL-H40055), ITGβ-1 levels in serum were measured. The detection range was 18.96–4000 pg/mL, and the sensitivity of the ITGβ-1 sandwich ELISA assay was 9.81 pg/mL. The assay showed no evidence of cross-reactivity or interference. Measurements were performed in duplicate to guarantee the accuracy and robustness of the results. The intra-assay CV was less than 5.5%, while the inter-assay precision was below 7.3%.

#### HIF-1α assay

The detection of human HIF-1α levels in the serum was performed with an ELISA kit (Elabscience Biotechnology Co. Ltd., USA, E-EL-H6066). The kit sensitivity for HIF-1α was 37.50 pg/mL, and the detection range was between 0.625 and 4000 pg/mL. No detectable cross-reactivity or interference was observed in this assay, and all measurements were replicated to ensure precision. The intra-assay variability was less than 6.5%, with inter-assay precision remaining under 7.4%.

#### MMP-2 and − 9 assays

The human MMP-2 ELISA kit (Elabscience Biotechnology Co. Ltd., USA, E-EL-H1445), a commercial kit, was used to determine the MMP-2 levels in the serum samples according to the test protocol. The human MMP-2 ELISA kit demonstrated a sensitivity of 0.47 ng/mL, with a detection range of 0.78 to 50 ng/mL. No cross-reactivity or interference was detected in the experiment. All the measurements were conducted in duplicate to enhance precision. The intra-assay and inter-assay CV were maintained at less than 3.6% and 4.6%, respectively.

The serum MMP-9 concentration was determined via an ELISA Human MMP-9 Kit (Elabscience Biotechnology Co. Ltd., USA, E-EL-H6075) according to the manufacturer’s instructions. The MMP-9 sandwich ELISA had a detection range of 1.6 to 10 ng/mL and a sensitivity of 0.10 ng/mL. The assay showed no evidence of cross-reactivity or interference. Duplicate of serum samples were used to guarantee the accuracy and robustness of the results. The CVs for the intra- and inter-assay measurements were kept below 4.4% and 5.0%, respectively.

### Statistical analysis

In determining the number of patients and controls to be included in the study, it was calculated that the sample size of the study should consist of at least 50 subjects for each group, taking GAL-3 as the parameter with the highest variation, Type I(α) = 0.05, and the power of the test as 0.85. The PASS 11 package program was used for this analysis.

The SPSS 27.0 statistical package program (SPSS Inc. Chicago, Illinois, USA) was used for statistical analysis of the data. Descriptive statistics are presented as arithmetic mean ± standard error values. The normality assumption of the continuous variables was examined with the Shapiro-Wilk normality test. The student’s t-test was applied to compare the means of two independent groups, and one-way analysis of variance (ANOVA) was used to compare more than two groups when the data were normally distributed. On the other hand, the Mann-Whitney U test was used as a non-parametric test when a normal distribution assumption was violated. The value of *p* < 0.05 was considered statistically significant in all the statistical analyses. Kendall’s tau-b correlation coefficient was used to evaluate the relationship between the serum levels of GAL-1, -3, -8, ITGβ-1, HIF-1α, MMP-2, and − 9.

Binary Logistic Regression analysis was performed to evaluate the diagnostic efficiency of the study variables for gliomas. GAL-1, -3, -8, ITGβ-1, HIF-1α, MMP-2, and − 9 were used as independent variables in the model. Significant variables were determined by calculating Odds Ratios (ORs) and 95% Confidence Intervals (CIs). Multinomial Logistic Regression analysis was applied to evaluate the impact of clinical parameters on glioma grade, histological tumor type, and localization and to identify important predictors of high malignant potential. Glioma grade, tumor type, and localization were coded as dependent variables, and clinical parameters were evaluated as independent variables. A forest plot was created to illustrate the ORs and 95% CIs for factors associated with sub-health based on logistic regression analysis. The diagnostic performance of parameters measured by ELISA in gliomas was evaluated using a receiver operating characteristic (ROC) curve analysis. During the analysis, the serum levels of GAL-1, -3, -8, ITGβ-1, HIF-1α, MMP-2, and − 9 from validated glioma patients were used as positive samples, whereas the serum from healthy individuals served as negative controls. Youden’s index was utilized as a criterion for determining the optimal cut-off point. The area under the curve (AUC) was considered statistically significant if its value exceeded 0.5.

## Results

Table 1 provides a detailed overview of the study group’s demographic characteristics along with the clinical and histopathologic features of the patients, including histologic glioma types, grades, tumor locations, Ki-67, and Glial Fibrillary Acidic Protein (GFAP) expressions (Table 1). The mean ages of the HGG, LGG, and control groups were 55.48 ± 1.51, 41.04 ± 1.66 and 39.20 ± 1.20 years, respectively, and their body mass indexes (BMIs) were 24.29 ± 0.52, 25.23 ± 0.33 and 25.82 ± 0.38, respectively. The HGG, LGG, and control groups consisted of 12 females and 38 males, 19 females and 31 males, and 16 females and 34 males, respectively.

Ki-67 is a cell proliferation marker, typically ranging from 15 to 40% in most glioblastomas (GBMs), and is predominantly observed in regions with high mitotic activity [23]. In our study, patients were categorized into three groups according to their Ki-67 proliferation index: ≤%5, between %5–15, and ≥%15.

**Table 1:**
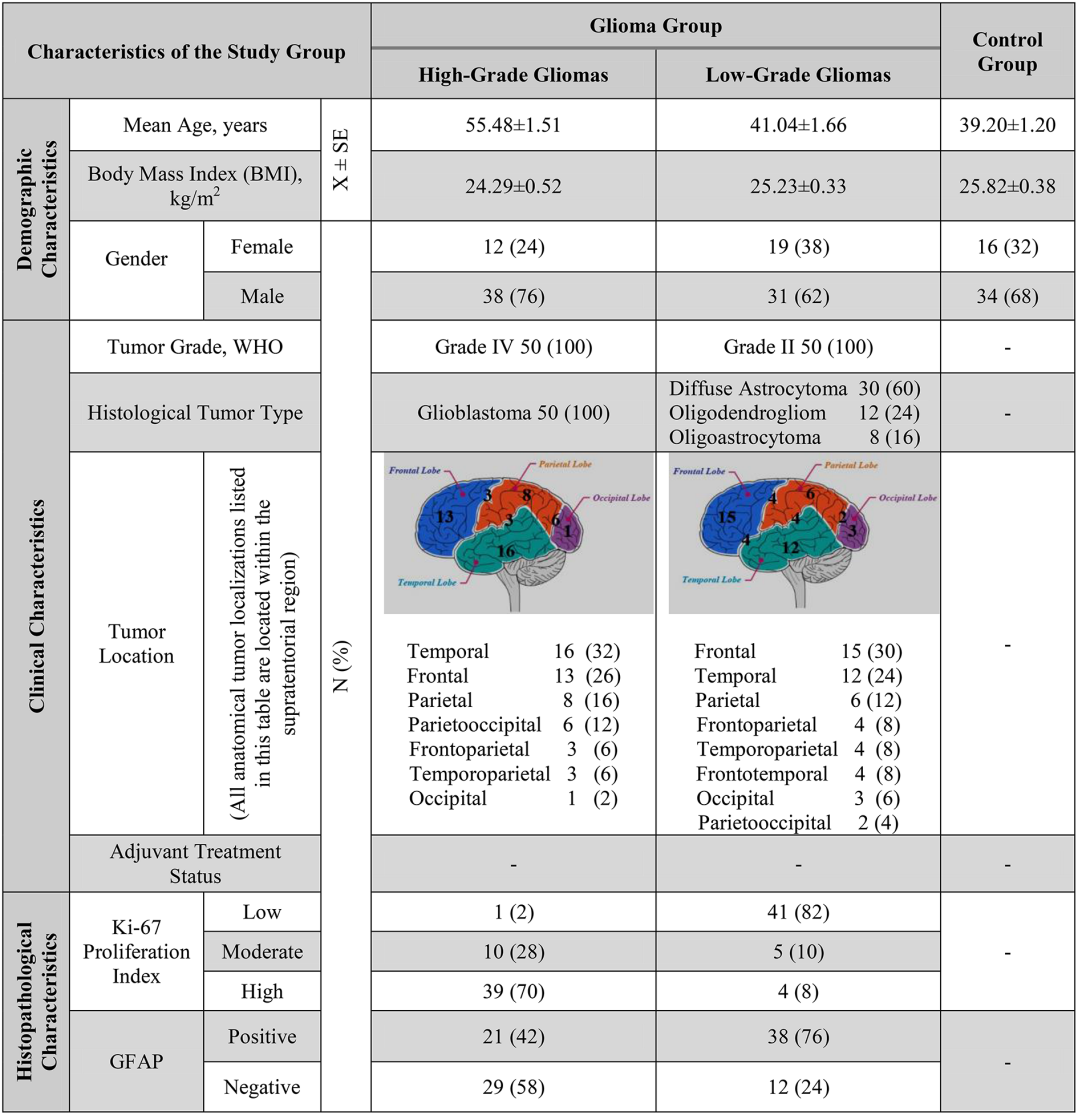
Characteristics of the study group

GFAP, widely utilized in histopathological and immunohistochemical diagnosis, is an intermediate filament protein synthesized by astrocytes and other cells of the CNS. As a key cytoskeletal component, GFAP contributes to cellular architecture and dynamics, regulates intercellular communication, and is crucial for the maintenance of blood-brain barrier (BBB) integrity and function. GFAP is considered an important marker of astrocytic differentiation and glial cell responses.

### GAL-1, -3 and − 8 levels

**Fig. 1:**
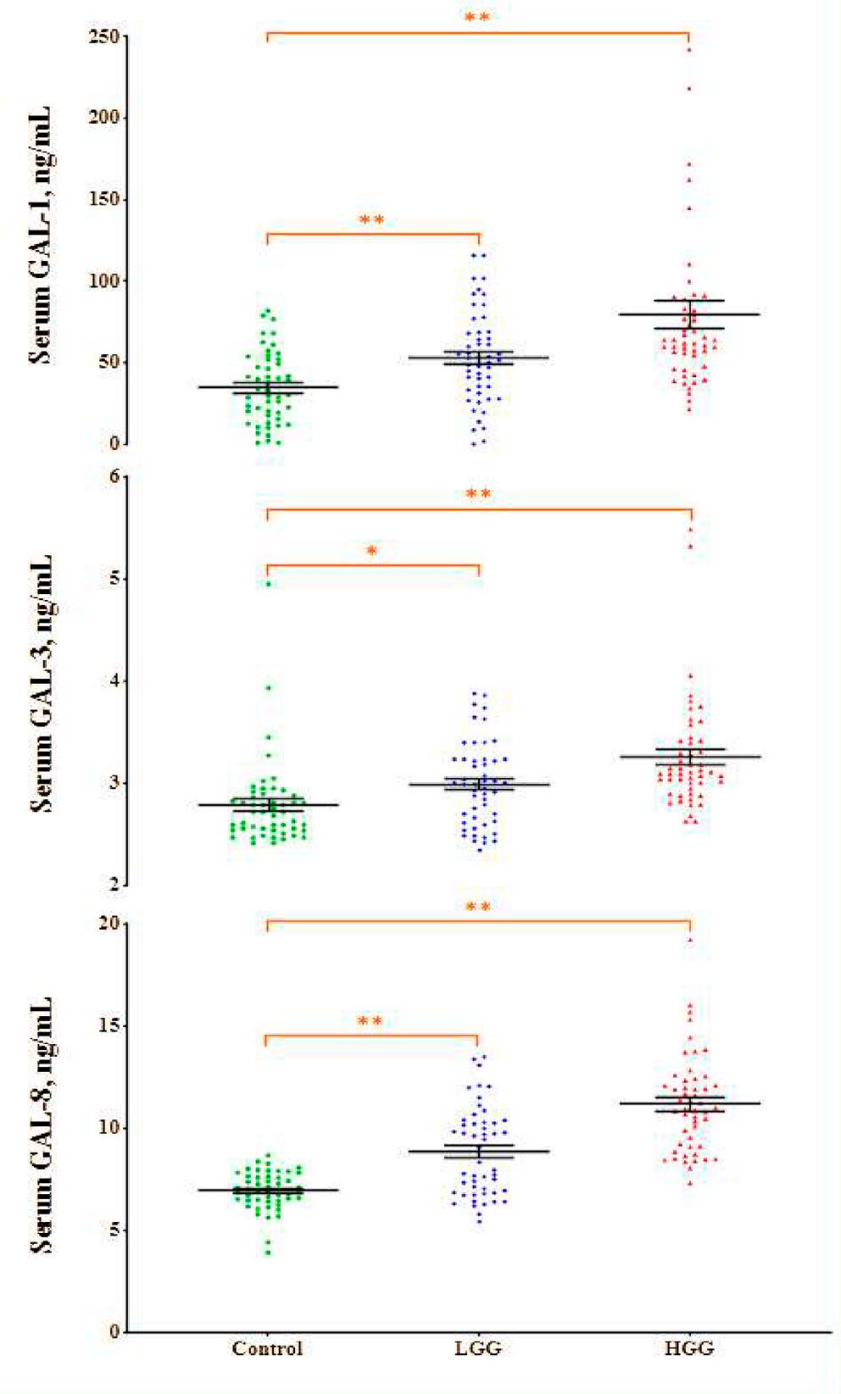
Serum levels of galectin family members GAL-1, GAL-3, and GAL-8 across control, LGG, and HGG groups. All three galectins exhibited a similar increasing trend from control to HGG. The long lines indicate the mean serum galectin levels, while the short lines represent ± standard deviations (± SDs). In all statistical analyses, the value of *p* < 0.05 was considered statistically significant. *Significant difference from the control group, *p* < 0.05. **Significant difference from the control group, *p* < 0.01

The serum GAL-1, -3, and − 8 levels in the HGG, LGG, and healthy control groups are given in Fig. 1. In the study, the serum GAL-1 levels were 79.80 ± 8.22 ng/mL in HGG, 53.18 ± 4.03 ng/mL in LGG, and 34.68 ± 3.06 ng/mL in the control group. Serum GAL-1 levels were significantly greater in both tumor groups compared to the healthy control group (*p* < 0.01, *p* < 0.01, respectively). Furthermore, the serum GAL-1 levels in the HGG group were determined to be significantly higher than that in the LGG group (*p* < 0.01).

Serum GAL-3 levels were measured as 3.25 ± 0.08 ng/mL in the HGG group, 2.99 ± 0.06 ng/mL in the LGG group, and 2.78 ± 0.06 ng/mL in the control group. Serum GAL-3 levels were significantly elevated in the HGG and LGG groups compared with those in the healthy control group (*p* < 0.05, *p* < 0.01, respectively). In addition, the HGG group exhibited significantly greater serum GAL-3 levels than did the LGG group (*p* < 0.01).

Serum GAL-8 levels were calculated as 11.19 ± 0.34 ng/mL in the HGG group, 8.84 ± 0.31 ng/mL in the LGG group, and 6.93 ± 0.13 ng/mL in the control group. Significant elevations in serum GAL-8 levels were observed in both tumor groups compared with those in the healthy group (*p* < 0.01, *p* < 0.01, respectively). Besides, serum GAL-3 levels in the HGG were higher compared to the LGG (*p* < 0.01).

### Serum ITGβ-1 levels

**Fig. 2:**
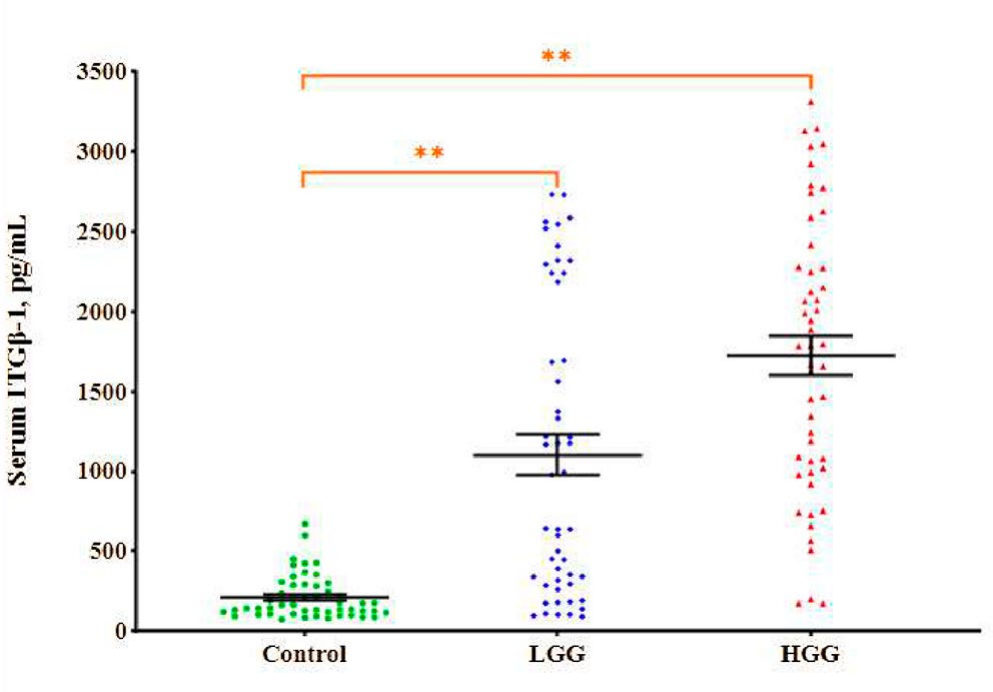
Serum ITGβ-1 levels in the study groups. The long lines indicate the mean serum ITGβ-1 levels, while the short lines represent ± SDs. In all statistical analyses, the value of *p* < 0.05 was considered statistically significant. **Significant difference from the control group, *p* < 0.01

Figure 2 presents the serum ITGβ-1 levels in HGG and LGG patients as well as in healthy individuals. The measured serum ITGβ-1 levels were 1723.81 ± 122.58 pg/mL in HGG, 1102.32 ± 129.45 pg/mL in LGG, and 209.55 ± 19.34 pg/mL in the control group. Significant differences in serum ITGβ-1 levels were observed between the glioma groups and the healthy group, with higher levels in both gliomas (*p* < 0.01, *p* < 0.01, respectively). Moreover, the serum ITGβ-1 levels in the HGG group were significantly raised than in the LGG group (*p* < 0.01).

### Serum HIF-1α levels

**Fig. 3:**
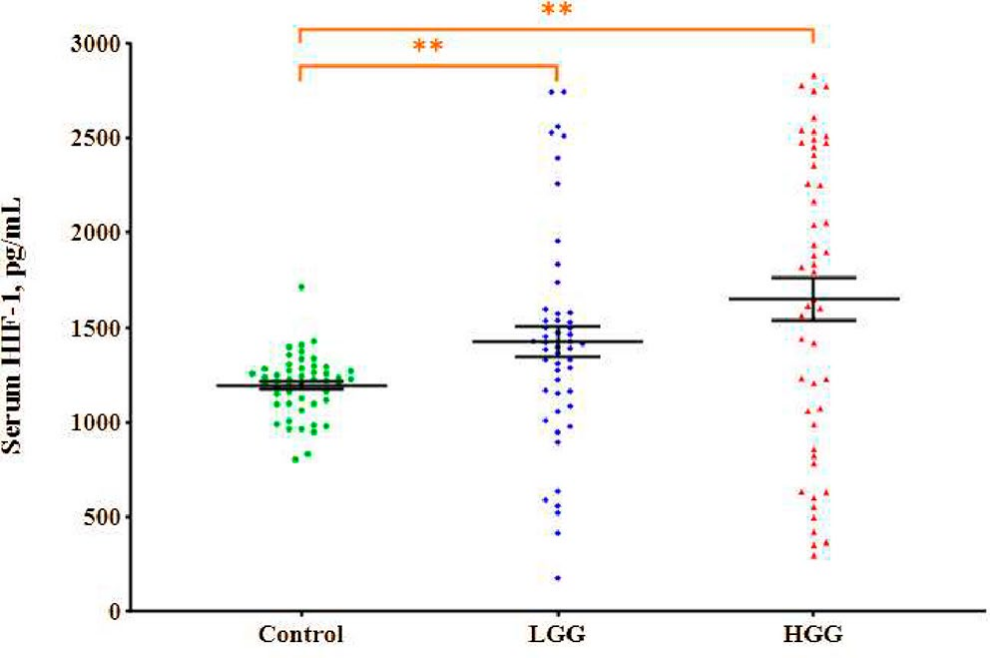
Serum HIF-1α levels in the study groups. The long lines indicate the mean serum HIF-1α levels, while the short lines represent ± SDs. In all statistical analyses, the value of *p* < 0.05 was considered statistically significant. **Significant difference from the control group, *p* < 0.01

Figure 3 illustrates the serum HIF-1α levels in patients with both HGG and LGG, along with those in the healthy control group. Analysis of the serum HIF-1α levels revealed values of 1647.43 ± 110.82 pg/mL in HGG patients, 1426.27 ± 81.96 pg/mL in LGG patients, and 1194.07 ± 22.86 pg/mL in the controls. Serum HIF-1α levels were significantly greater in HGG and LGG patients than in the healthy ones (*p* < 0.01, *p* < 0.01, respectively). No significant difference was found in the serum HIF-1α levels between HGG and LGG patients (*p* > 0.05).

### Serum MMP-2 and − 9 levels

**Fig. 4:**
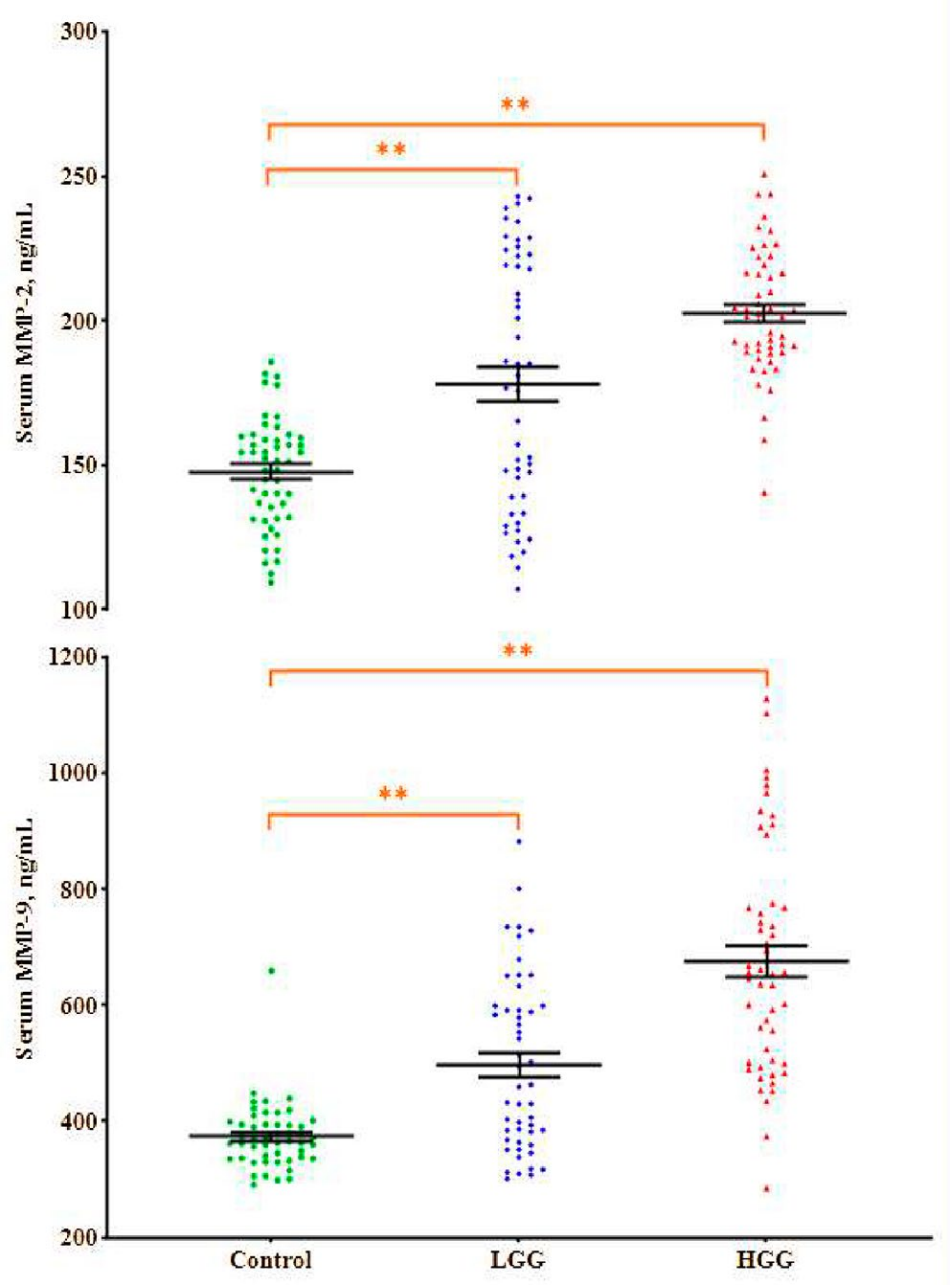
Serum levels of MMP family members MMP-2 and MMP-9 across control, LGG, and HGG groups. Both matrix metalloproteinases exhibited a similar increasing trend from control to HGG. The long lines indicate the mean serum matrix metalloproteinase levels, while the short lines represent ± SDs. In all statistical analyses, the value of *p* < 0.05 was considered statistically significant. *Significant difference from the control group, *p* < 0.05. **Significant difference from the control group, *p* < 0.01

Figure 4 shows the serum MMP-2 and − 9 levels of HGG, LGG, and healthy controls. Serum MMP-2 levels were identified as 202.42 ± 3.11 ng/mL in the HGG, 178.16 ± 6.13 ng/mL in the LGG, and 147.61 ± 2.67 ng/mL in the control group. Compared with the controls, both patient groups showed statistically significant increases in serum MMP-2 levels (*p* < 0.01, *p* < 0.01, respectively). Additionally, the serum MMP-2 levels in the HGG tumor group were statistically greater than those in the LGG tumor group (*p* < 0.01).

The findings revealed serum MMP-9 levels of 675.29 ± 27.95 ng/mL in HGG, 497.15 ± 21.27 ng/mL in LGG, and 373.37 ± 8.07 ng/mL in the control ones. HGG and LGG patients displayed significantly elevated serum MMP-9 levels compared to healthy individuals (*p* < 0.01, *p* < 0.01, respectively). Moreover, serum MMP-9 levels were higher in HGG than in LGG (*p* < 0.01).

Using bivariate correlation analysis among measured parameters in HGG, significant positive correlations were revealed between GAL-8 and ITGβ-1 (*p* < 0.01), whereas a negative correlation was detected between GAL-8 and MMP-2 (*p* < 0.05).

In LGG, a series of positive correlations were identified, including between GAL-1 and GAL-3 levels (*p* < 0.01), GAL-3 and GAL-8 (*p* < 0.01), GAL-3 and ITGβ-1 (*p* < 0.01), GAL-3 and MMP-2 (*p* < 0.01), GAL-3 and MMP-9 (*p* < 0.01), GAL-8 and ITGβ-1 (*p* < 0.01), GAL-8 and MMP-2 (*p* < 0.01), GAL-8 and MMP-9 (*p* < 0.01), ITGβ-1 and MMP-2 (*p* < 0.01), ITGβ-1 and MMP-9 (*p* < 0.01), MMP-2 and MMP-9 (*p* < 0.01).

In the control group, GAL-3 and GAL-8 levels were positively correlated (*p* < 0.05). On the other hand, negative correlations were found between GAL-1 and MMP-9 (*p* < 0.05).

### Logistic regression analysis

Variables demonstrating a statistically significant difference in the univariate analysis were incorporated as independent variables in the binary logistic regression analysis. Seven independent predictors were identified as influencing diagnostic efficacy in glioma patients. The p value of Omnibus tests of our model was ≤ 0.001, the Nagelkerke R Square was 0.886, and the Hosmer and Lemeshow Test was 0.995, demonstrating that our model has statistically significant differences and better goodness of fit. According to the model, the accuracy of glioma diagnosis efficacy was 95.9%. Serum levels of GAL-1 (OR: 1.052, 95% CI: 1.008–1.098, *p* < 0.05), GAL-8 (OR: 1.890, 95% CI: 1.111–3.215, *p* < 0.05), ITGβ-1 (OR: 1.017, 95% CI: 1.007–1.027, *p* < 0.01), HIF-1α (OR: 1.016, 95% CI: 1.006–1.025, *p* < 0.01), and MMP-9 (OR: 1.023, 95% CI: 1.003–1.044, *p* < 0.05) were positively related to diagnostic efficacy and were identified as independent risk factors for glioma. Conversely, GAL-3 and MMP-2 levels were not directly associated with the presence of glioma. With an OR of 1.890, GAL-8 has emerged as one of the most strongly associated biomarkers with glioma presence. Details of the binary logistic regression analysis are summarized in Table 2.

**Table 2:** Binary logistic regression analysis of the study variables on glioma affecting diagnostic efficiency

Study Variables	β	SE	Wald	*p* Value	OR	95% CI of OR
**GAL-1**	0.051	0.022	5.506	**0.019**	1.052	1.008–1.098
**GAL-3**	-1.439	1.684	0.730	0.393	0.237	0.009–6.435
**GAL-8**	0.636	0.271	5.509	**0.019**	1.890	1.111–3.215
**ITGβ1**	0.017	0.005	10.551	**0.001**	1.017	1.007–1.027
**HIF1α**	0.015	0.005	9.865	**0.002**	1.016	1.006–1.025
**MMP-2**	0.015	0.021	0.546	0.460	1.015	0.975–1.057
**MMP-9**	0.023	0.010	5.249	**0.022**	1.023	1.003–1.044
**Constant**	-38.620	12.372	9.744	0.002	0.000	

β: Beta coefficient, SE: Standard Error, OR: Odds Ratio, 95% CI: 95% confidence intervals, bold: Significant Differences, The p value of Omnibus tests was < 0. 001, the Nagelkerke R^2^ was 0.886 and Hosmer and Lemeshow Test was 0.995

In this study, both ordinal and multinomial logistic regression models were applied to evaluate the effects of clinical parameters on glioma grade. As a result of the comparisons, the multinomial logistic regression model, which yielded a lower AIC value, was determined to be more appropriate for the data, and the parameter estimates for this model are presented in Table 3. According to the results of the multinomial logistic regression analysis, GAL-1, GAL-8, and MMP-9 did not have a significant effect on LGGs but had a significant effect on HGGs, indicating that these proteins are important biomarkers that can be evaluated, especially in the differentiation of HGG from LGG. Among these proteins, GAL-8 was the most potent biomarker for determining glioma grade and malignant progression (OR: 1.911, 95% CI: 1.175–3.110, *p* < 0.01). Similarly, ITGβ-1 and HIF-1α were also associated with both HGG and LGG, suggesting that these proteins may be promising markers that may help differentiate LGGs from healthy individuals. On the other hand, GAL-3 and MMP-2 had no significant effect on glioma grade.

**Table 3:** Multinomial logistic regression analysis of the study variables on gliomas affecting grade

Grade*	Study Variables	β		SE	Wald	*p* Value		OR	95% CI of OR
**II**	**Intercept**	-15.007		4.841	9.610	**0.002**			
	**GAL-1**	0.018		0.013	1.930	0.165		1.018	0.993–1.045
	**GAL-3**	-0.335		0.936	0.128	0.720		0.715	0.114–4.477
	**GAL-8**	0.206		0.248	0.690	0.406		1.229	0.756–1.999
	**ITGβ1**	0.006		0.002	10.935	**< 0.001**		1.006	1.003–1.010
	**HIF1α**	0.006		0.002	10.962	**< 0.001**		1.006	1.002–1.010
	**MMP-2**	0.007		0.014	0.281	0.596		1.007	0.980–1.035
	**MMP-9**	0.007		0.005	1.921	0.166		1.007	0.997–1.016
**IV**	**Intercept**	-26.261		5.724	21.051	**< 0.001**			
	**GAL-1**	0.040		0.016	5.984	**0.014**		1.040	1.008–1.074
	**GAL-3**	-0.010		1.072	0.000	0.992		0.990	0.121–8.094
	**GAL-8**	0.648		0.248	6.802	**0.009**		1.911	1.175–3.110
	**ITGβ1**	0.006		0.002	9.902	**0.002**		1.006	1.002–1.010
	**HIF1α**	0.006		0.002	11.908	**< 0.001**		1.006	1.003–1.010
	**MMP-2**	0.013		0.017	0.584	0.445		1.013	0.980–1.048
	**MMP-9**	0.012		0.005	5.612	**0.018**		1.012	1.002–1.022
**Pseudo R-Square**									
**Cox and Snell**			**Nagelkerke**				**McFadden**		
**0.680**			**0.765**				**0.518**		

*The reference category is the healthy group. β: Beta coefficient, SE: Standard Error, OR: Odds Ratio, 95% CI: 95% confidence intervals, bold: Significant Differences, The p value of Final Model Fitting Information was < 0. 001

According to multinominal logistic regression analysis, with healthy controls used as the reference category, we also assessed the impact of clinical parameters on histological glioma type and found that the increase in serum GAL-8 levels (OR: 2.167, 95% CI: 1.126–4.172, *p* < 0.05) was a risk factor for GBM. Furthermore, the increase in serum GAL-8 levels was strongly associated with a 1.627-fold greater (95% CI: 0.992–2.669, *p* < 0.05) likelihood of glioma formation, particularly in the temporoparietal region.

**Fig. 5:**
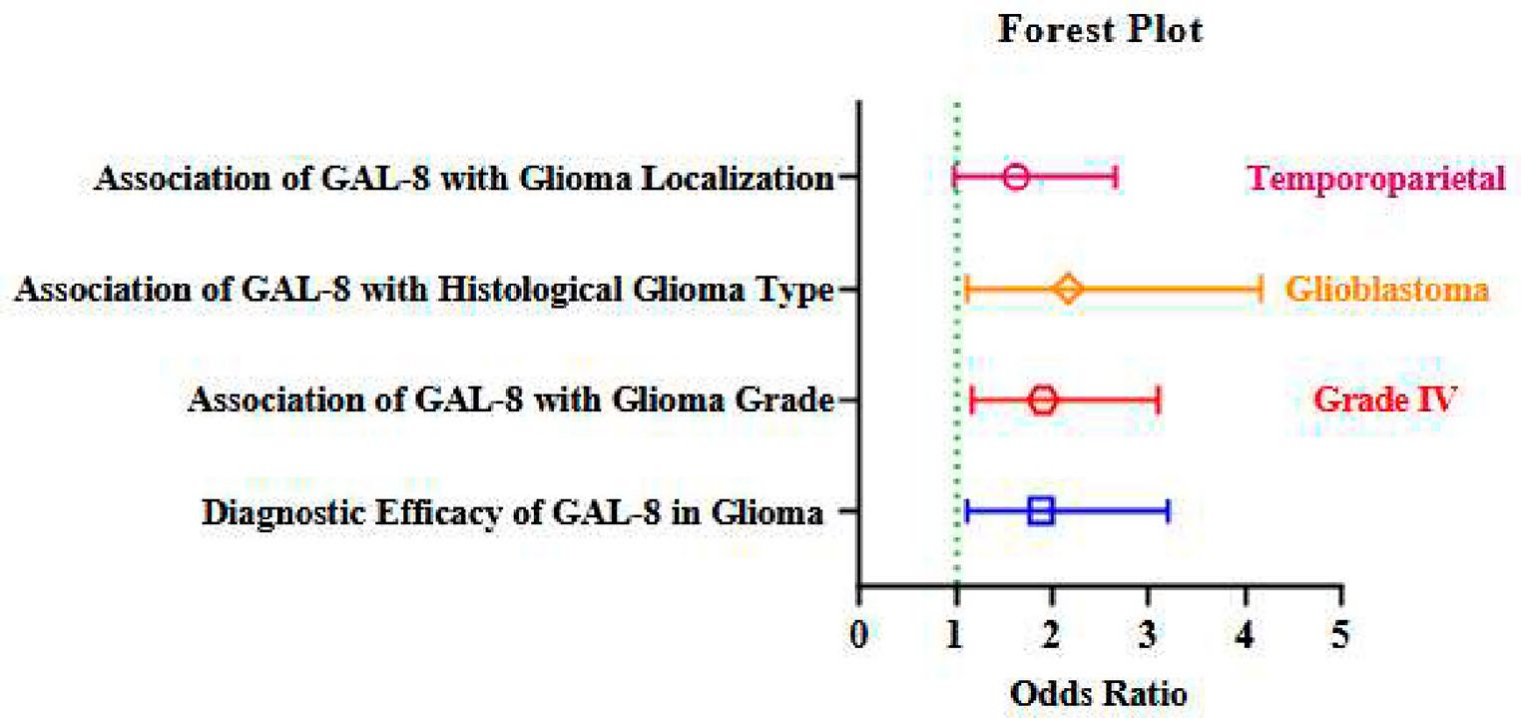
Forest plot of the effects of Gal-8 on Glioma formation, progression, histological type, and localization Note: All Grade IV gliomas in this analysis correspond to glioblastomas; however, both grade and histological subtype were analyzed separately to reflect distinct clinicopathological dimensions within the cohort

The forest plot based on binary and multinomial logistic regression analyses showed that GAL-8 influences diagnostic efficacy in glioma (OR: 1.890, 95% CI: 1.111–3.215, *p* < 0.05), glioma grade and malignant progression (OR: 1.911, 95% CI: 1.175–3.110, *p* < 0.01), histological glioma type (OR: 2.167, 95% CI: 1.126–4.172, *p* < 0.05), and glioma localization (OR: 1.627, 95% CI: 0.992–2.669, *p* < 0.05) in the final regression model (Fig. 5).

**Fig. 6:**
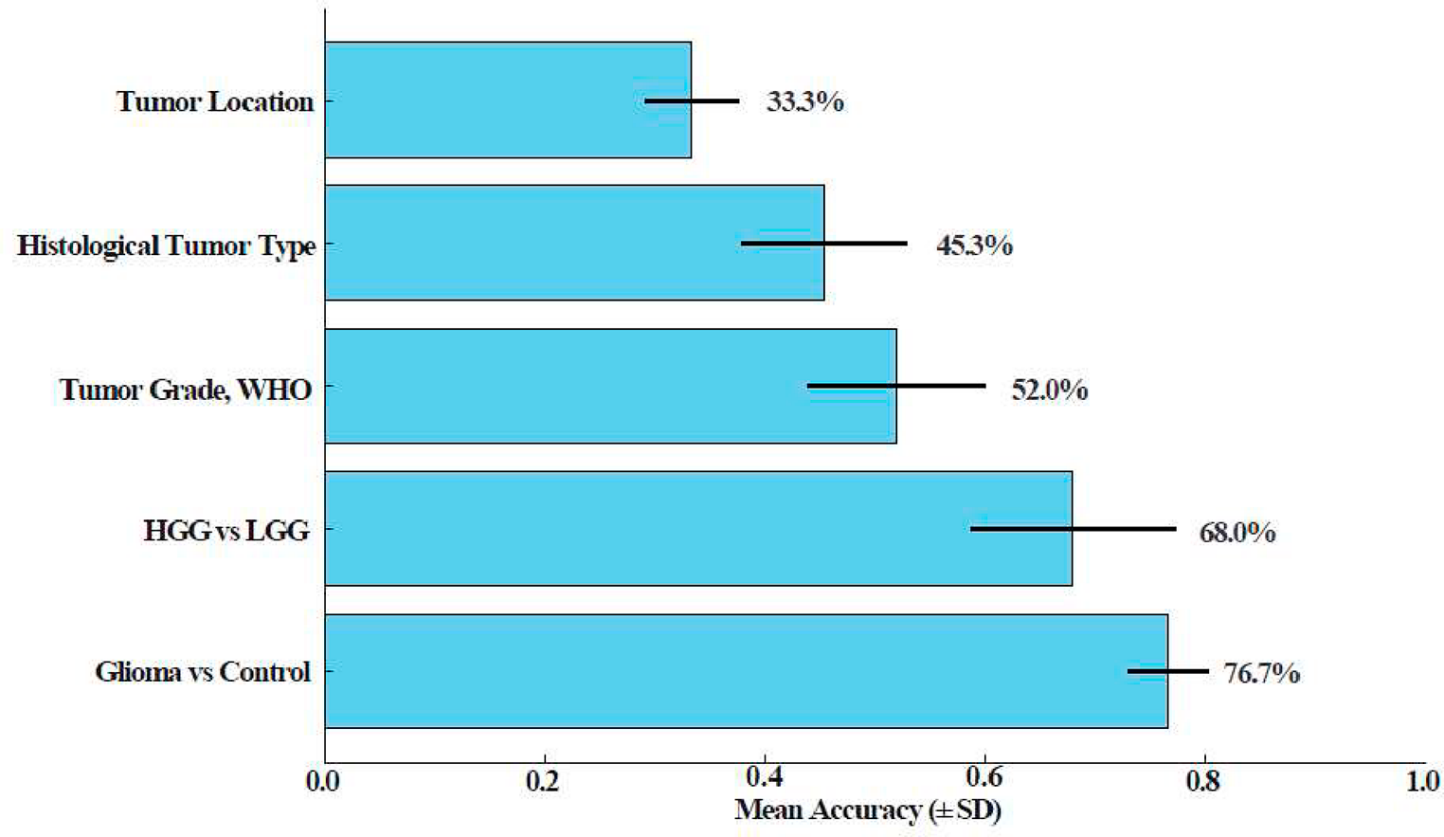
Diagnostic performance of serum GAL-8 for glioma classification using 5-fold cross-validation. Mean classification accuracy and standard deviation are shown for each task

5-fold cross-validation analysis was also conducted using GAL-8 serum levels to evaluate its diagnostic performance. The results demonstrated strong classification accuracy for distinguishing glioma patients from healthy controls with the mean accuracy of 76.7%±3.7% and moderate performance for differentiating HGGs from LGGs with a mean accuracy of 68.0%±9.3%. On the other hand, the performance of GAL-8 in predicting tumor grade, histological tumor subtype, and localization was limited. (Fig. 6).

### ROC analysis

**Fig. 7:**
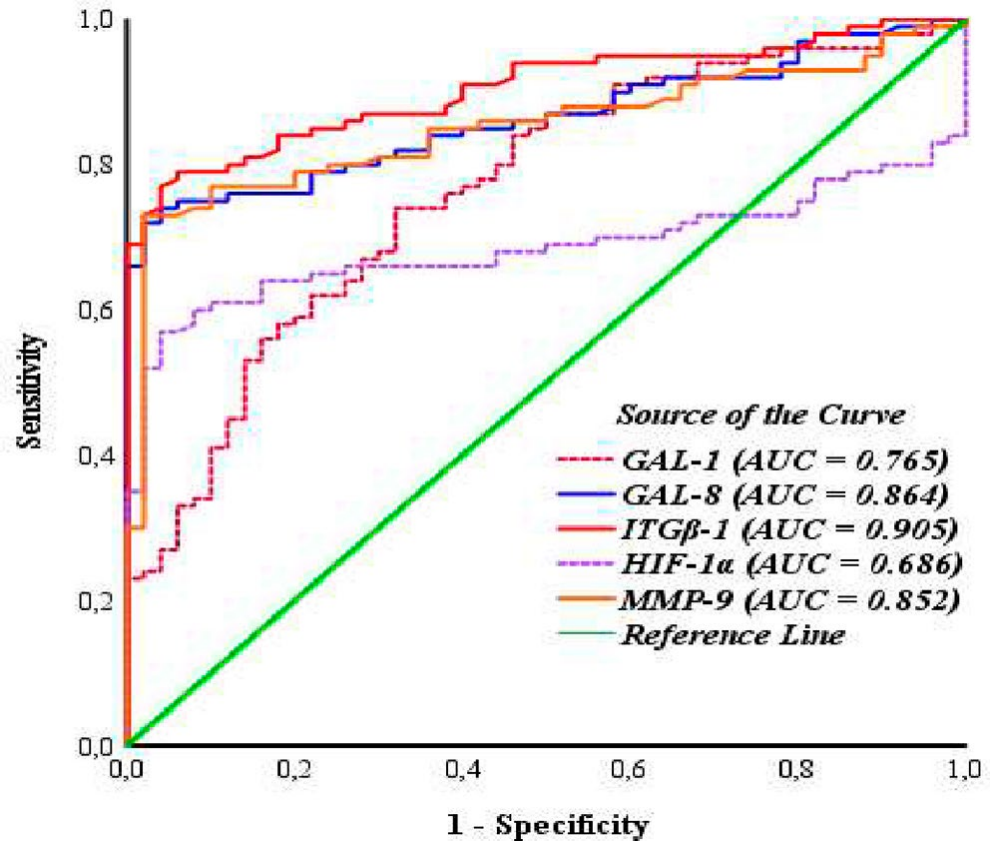
ROC curves for serum GAL-1, GAL-8, ITGβ-1, HIF-1α and, MMP-9 in glioma

The diagnostic values of serum GAL-1, GAL-8, ITGβ-1, HIF-1α, and MMP-9 as a potential biomarker for gliomas were assessed using ROC curve analysis. In glioma patients, the AUCs for serum GAL-1, GAL-8, ITGβ-1, HIF-1α, and MMP-9 were 0.765 [95% CI (0.686–0.843), *p* < 0.01], 0.864 [95% CI (0.807–0.921), *p* < 0.01], 0.905 [95% CI (0.858–0.951), *p* < 0.01], 0.686 [95% CI (0.601–0.770), *p* < 0.01], and 0.852 [95% CI (0.791–0.913), *p* < 0.01], respectively. The diagnostic sensitivity values of these proteins were 74%, 74%, 79%, 57%, and 73%, while their corresponding specificity values were 68%, 96%, 94%, 96%, and 98%, respectively. The results of the ROC analysis are illustrated in Fig. 7.

In the ROC analysis, the threshold values of the serum GAL-1, GAL-8, ITGβ-1, HIF-1α, and MMP-9 levels for diagnosing glioma were 41.83 ng/mL, 8.28 ng/mL, 436.34 pg/mL, 1411.00 pg/mL, and 450.08 ng/mL, respectively.

Using this cut-off point, we subsequently analyzed the link between the serum levels of the measured parameters and glioma via bivariate analysis. A total of 34 healthy controls and 26 glioma patients had GAL-1 levels below the cut-off value, whereas 16 healthy controls and 74 glioma patients were observed above this threshold. The results revealed that individuals whose serum GAL-1 levels > 41.83 ng/mL were significantly associated with the presence of glioma than those whose GAL-1 levels below 41.83 ng/mL (OR: 6.05, *p* < 0.01).

When GAL-8 levels were below the cut-off value; of 8.28 ng/mL, 48 controls and 26 patients were identified, compared to 2 controls and 74 patients with levels exceeding the cut-off value for GAL-8. Persons whose serum GAL-8 exceeding 8.28 ng/mL exhibited a strong association with glioma diagnosis compared to those with lower (OR: 68.31, *p* < 0.01).

In the study cohort, ITGβ-1 levels were detected below the cut-off value in 47 healthy individuals and 21 glioma patients, while 3 healthy individuals and 79 patients had levels exceeding this threshold. Compared with lower levels, a serum ITGβ-1 concentration above 436.34 pg/mL was strongly correlated with glioma status (OR: 58.94, *p* < 0.01).

Among the study participants, HIF-1α levels were below the cut-off value in 48 controls and 43 patients, though 2 controls and 57 patients exhibited HIF-1α levels above this threshold. Individuals whose serum HIF-1α concentration surpassed 1411.00 pg/mL demonstrated statistically elevated probability of developing glioma than did those with lower ones (OR: 31.81, *p* < 0.01).

MMP-9 levels fell below the cut-off value in 49 healthy subjects and 27 glioma patients. However, 1 healthy subject and 73 glioma patients showed higher MMP-9 levels than the threshold. Elevated serum MMP-9 values > 450.08 ng/mL were strongly associated with glioma cases compared to lower values (OR: 132.48, *p* < 0.01).

However, these associations should be interpreted with caution given the retrospective design and limited sample size. Prospective, multicenter studies are warranted to validate these threshold-based findings.

**Fig. 8:**
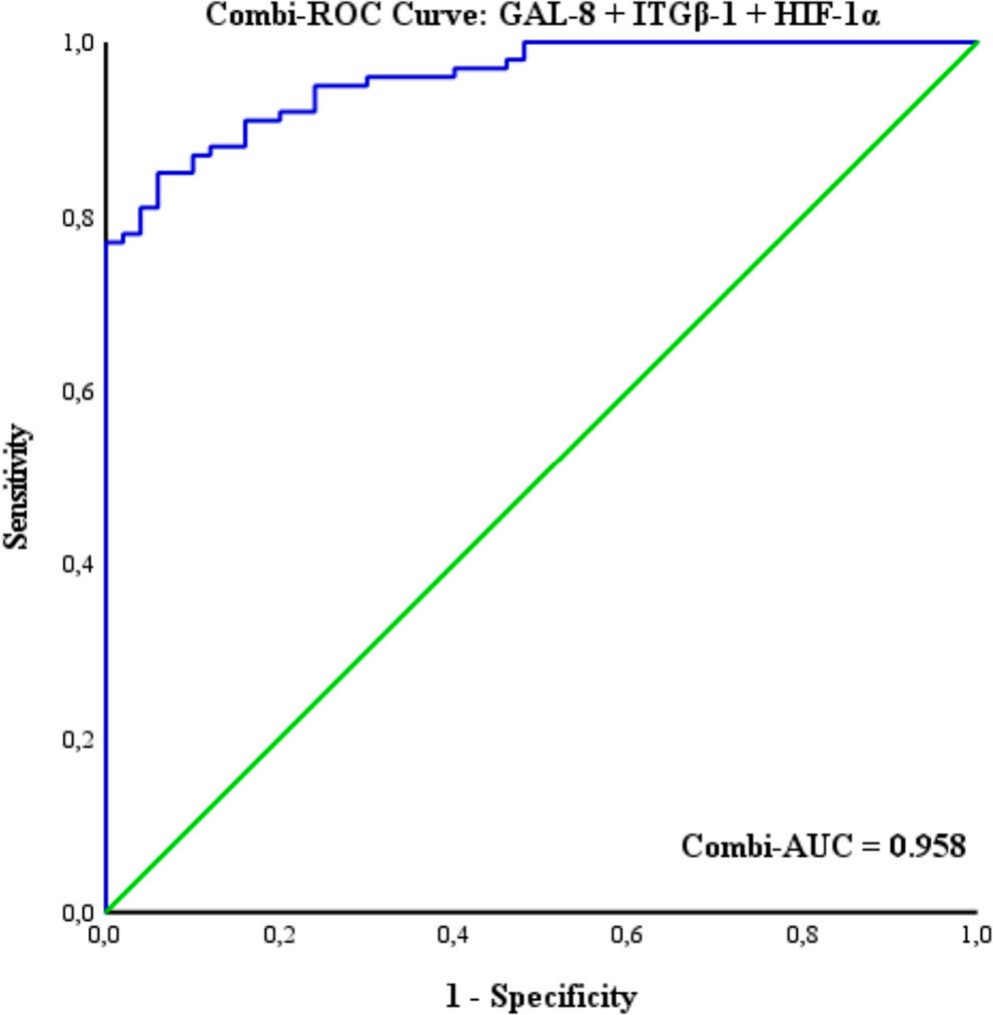
Combi-ROC Curve: GAL-8 + ITGβ-1 + HIF-1α

The combined biomarker panel demonstrated excellent diagnostic performance, with an AUC of 0.959, suggesting a potential additive or synergistic interaction among GAL-8, ITGβ-1, and HIF-1α (Fig. 8). At the optimal classification threshold of 0.595, the panel achieved a sensitivity of 85% and a specificity of 94%, highlighting its strong potential for discriminating glioma patients from healthy controls.

**Fig. 9:**
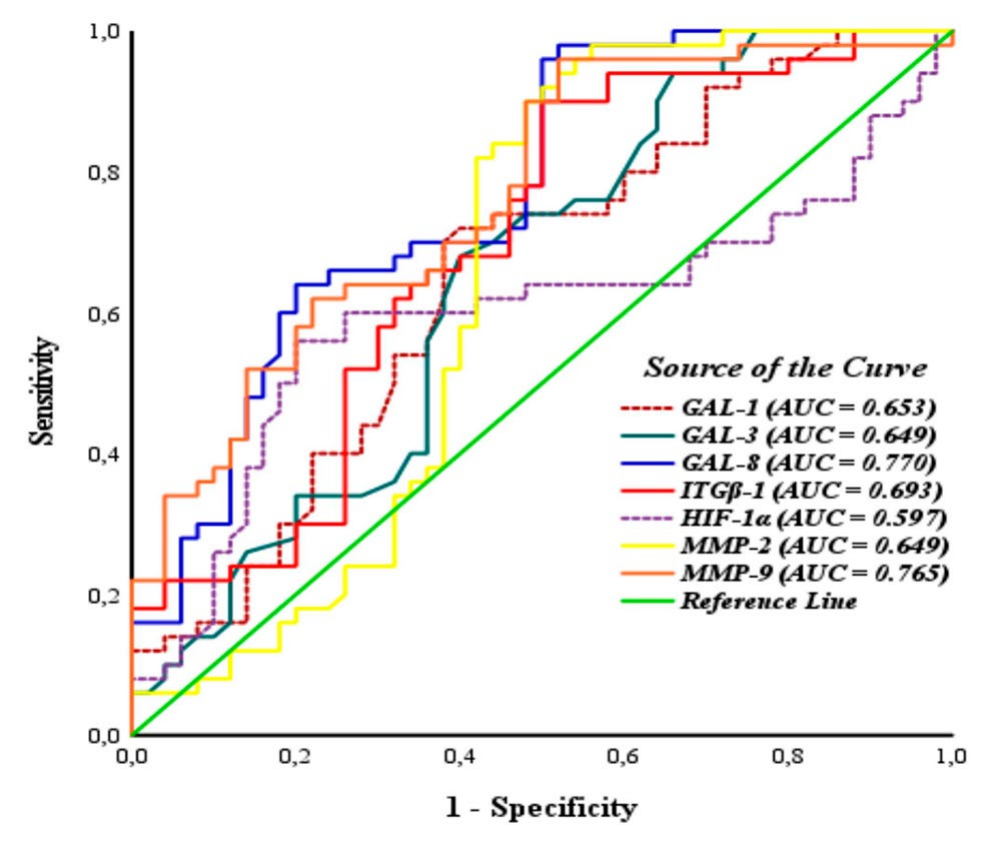
ROC curves for serum biomarkers in distinguishing LGG from HGG

The diagnostic values of serum GAL-1, GAL-3, GAL-8, ITGβ-1, HIF-1α, MMP-2 and MMP-9 as a potential biomarker in distinguishing LGG from HGG were assessed using ROC curve analysis. In HGG patients, the AUCs for serum GAL-1, GAL-3, GAL-8, ITGβ-1, HIF-1α, MMP-2, and MMP-9 were 0.653 [95% CI (0.546–0.761), *p* < 0.01], 0.649 [95% CI (0.541–0.758), *p* < 0.05], 0.770 [95% CI (0.679–0.862), *p* < 0.01], 0.693 [95% CI (0.589–0.797), *p* < 0.01], 0.597 [95% CI (0.480–0.713), *p* > 0.05], 0.649 [95% CI (0.533–0.764), *p* < 0.05], and 0.765 [95% CI (0.673–0.857), *p* < 0.01], respectively. The diagnostic sensitivity values of these proteins were 70%, 68%, 96%, 90%, 56%, 90% and 96%, while their corresponding specificity values were 62%, 60%, 50%, 50%, 80%, 52% and 48%, respectively. The results of the ROC analysis are illustrated in Fig. 9. GAL-8 and MMP-9 have the highest AUC values ​​in distinguishing LGG from HGG.

## DISCUSSION

Gliomas are the most lethal tumors, with the highest percentage and fastest progression among primary brain tumors [24]. HGGs are quite aggressive and the survival time of patients is very short. The median survival time from the moment of diagnosis of glioma is usually less than one year, and even under the best conditions, only 5–10% of patients survive up to 2 years [25, 26]. LGGs gradually transform into HGGs due to the lack of a complete treatment option. For this reason, the number of studies aimed at determining the molecular pathogenesis of gliomas, and using this information in the development of new treatment methods has increased in recent years. In light of current knowledge, although the angiogenic activities of HGGs are exceptionally high, the key mechanisms involved in this activity have not yet been fully elucidated. Following the updated classification of gliomas by Louis et al. in 2021 [27], taking into account the role of molecular diagnosis as well as histological features and immunohistochemistry, studies on the identification of biomarkers that may allow early diagnosis in gliomas of different grades and histological types and new molecular targets that may provide alternative methods for treatment have accelerated. The identification of easily accessible serum biomarkers to aid in the diagnosis and prognosis of gliomas is an urgent and unmet need in the field of neuro-oncology.

Galectins are multifunctional proteins involved in key biological processes that support tumor survival and progression, including cell proliferation, apoptosis, transcriptional regulation, intracellular signaling, cell adhesion, and migration. Studies in different cancer models have shown that the presence of GAL-1 in the tumor microenvironment significantly contributes to tumor progression by suppressing the local immune response and thus promoting angiogenesis, tumor cell migration, and chemotherapy resistance [28]. However, studies investigating the relationship between serum GAL-1 levels and gliomas are quite limited in the literature. In a study conducted by Verschuere et al., the diagnostic potential of serum GAL-1 levels in glial tumors was evaluated and it was reported that in newly diagnosed GBM patients, age- and sex-adjusted serum GAL-1 levels were significantly higher compared to healthy controls [29]. In addition, the same study reported that GAL-1 levels did not significantly decrease during follow-up after surgical resection. This suggests that GAL-1 may be related not only to the presence of the tumor but also to the dynamics of the tumor microenvironment and immune response. It was also found that GAL-1 levels were significantly higher in patients with relapsed HGGs compared to healthy controls. When the diagnostic power of serum GAL-1 levels was evaluated by ROC analysis, a high AUC value of 0.968 was found for distinguishing GBM patients from healthy individuals, and concluded that the diagnostic accuracy of GAL-1 increases with age. These findings are consistent with the increased serum GAL-1 levels in HGGs and LGGs compared with those in healthy controls in our study, supporting the view that GAL-1 may play an important role in the biological behavior of gliomas. The fact that serum GAL-1 levels are significantly higher in HGGs than in LGGs increases the potential of serum GAL-1 levels as a prognostic marker for the diagnosis and follow-up of gliomas in older age groups. In our study, the area under the ROC curve for serum GAL-1 levels was 0.765, and the risk of glioma was found to be 6.05 times greater at concentrations above the cut-off value. Yamaoka et al. examined the mRNA expression of GAL-1 in 12 different glioma cell lines and 27 glioma tissues, 9 astrocytoma, 2 anaplastic astrocytoma, and 16 GBM [30]. In this study, very low basal GAL-1 mRNA levels were shown in normal glia, whereas significantly higher levels were detected in glioma tissue samples. GAL-1 mRNA levels were much higher in GBMs compared to low-grade astrocytomas. Specifically, it has been reported that the relative GAL-1 mRNA levels are 3.7-fold higher in low-grade astrocytoma, 5.8-fold greater in anaplastic astrocytoma, and 6.4-fold higher in GBM compared to normal glial tissue. In addition to its elevated expression in tumor specimens, GAL-1 has also been detected at high levels in glioma cell lines, except for the U343MG cell line. Enhanced GAL-1 expression has been reported in HGGs, and suggested that this may be related to the tumor stage. Similar to the findings of the study by Yamaoka et al., serum GAL-1 levels were higher in grade IV glial tumors than in grade II tumors. These observations demonstrate a strong correlation between GAL-1 levels and tumor grade, reinforcing its potential role in glioma pathophysiology. Likewise, according to histological tumor type, the highest serum GAL-1 levels were found in GBM patients compared with those in other gliomas, which is consistent with the tissue expression levels obtained by Yamaoka et al. In another study conducted to reveal the roles of GAL-1 in glial tumor biology, malignant glioma cells with high infiltrative capacity presented higher GAL-1 expression compared to non-invasive regions [31]. The overexpression of GAL-1 in the U87MG cell line increased the migration and invasion capacities of the cells in vitro, while in vivo models, it caused an increase in tumor invasion and decreased survival. The authors suggested that GAL-1 may be a critical regulator of glial tumor cell migration, invasion, neoangiogenesis, and immune system evasion mechanisms and that therapeutic strategies targeting GAL-1 are a potential approach that may provide clinical benefit in addition to current treatments. In another study evaluating the prognostic significance of GAL-1 in GBM patients receiving adjuvant RT, Chou et al. reported that the median survival of patients with low GAL-1 expression was significantly longer than those with high expression [32]. It was concluded that the GAL-1 expression level may be an important biomarker affecting survival after adjuvant RT in GBM patients. Le Mercier et al. investigated the effect of GAL-1 on chemotherapy resistance in GBM cells and demonstrated that hypoxia-induced GAL-1 increased tumor cell migration and angiogenesis [33]. The findings suggest that GAL-1 plays a critical role in GBM pathophysiology and has the potential to be evaluated as a therapeutic target. Our study extends all these findings in the literature by providing additional information on the impact of GAL-1 on tumor progression and patient prognosis. Considering the high serum levels of GAL-1 in HGGs, GAL-1 may serve as a potential biomarker for disease severity and a target for therapeutic interventions. Furthermore, investigating its interaction with other circulating biomarkers may provide a more comprehensive understanding of glioma pathogenesis and molecular heterogeneity.

GAL-3 has been shown to be a multifunctional protein involved in various biological processes, including cell-cell and cell-matrix interactions, cell adhesion, cell activation, cell growth, differentiation, cell cycle regulation, apoptosis, and angiogenesis, among many other cellular functions [34, 35]. Although there are no studies in the literature evaluating GAL-3 serum levels in gliomas, there are studies showing increases in different cancers, such as gastric, hepatocellular, ovarian, pancreatic, and colorectal, compared with controls [36–40]. On the other hand, studies have investigated the expression of GAL-3 in human glial tumor cell lines and glial tissues. In one of these studies, Camby et al. examined the expression levels of galectins in human astrocytic tumors and their roles in tumor invasion [13]. In the study, GAL-1, -3, and − 8 levels were examined via immunohistochemistry in 116 human astrocytic tumor samples, and the transcription levels of galectin genes were determined using RT-PCR in 8 different human GBM cell lines. It has been reported that GAL-1 and − 3 expression significantly change with the malignancy progression in human astrocytic tumors, but GAL-8 expression remains unchanged. Researchers have determined that GAL-1, -3, and, to a lesser extent, -8 play a role in the invasion of GBMs into the brain parenchyma. In GBM, a well-established observation is that elevated GAL-3 expression is correlated with worse prognosis and increased tumor aggressiveness [41–44]. In our study, the serum GAL-3 level of the glioma patient group was greater than the healthy control group. Similarly, the positive correlation between serum GAL-3 levels and tumor grade was confirmed by the obtained results.

GAL-8 regulates key cellular processes associated with cell adhesion, migration, proliferation, and survival [45]. Very few studies have focused on the relationship between GAL-8 and glioma pathogenesis, and no studies have evaluated the serum levels of it in glial tumors. However, there are conflicting results evaluating serum GAL-8 levels in various cancers. A study conducted by Barrow et al. reported that serum GAL-8 levels were significantly higher in colon and breast cancer patients than in healthy individuals and were 1.8-fold higher [46]. In the same study, circulating GAL-8 levels were found to be 5.6-fold higher in patients with metastasis than in those without metastasis. In support of the study by Barrow et al., GAL-8 is considered an indicator of poor prognosis in various tumor types, including cervical, breast, ovarian, prostate, and metastatic melanoma [47–51]. On the other hand, another study reported that the cellular expressions of GAL-8 were lower in colorectal cancer than in healthy colon epithelium [52]. The role of GAL-8 in glioma progression and whether it is a therapeutic target is not yet clear. To date, only a few studies have directly addressed these questions. The first of these studies was performed by Metz et al., and the effects of GAL-8 on migration, proliferation, and apoptosis were examined in the U87 GBM cell model [22]. This study showed for the first time that GAl-8 increased proliferation in glial cells, prevented apoptosis, and contributed to the excessive malignancy potential of GBM cells by promoting cell proliferation, survival, and migration abilities and inhibiting apoptosis in these cells. However, it is not known whether these observations were confirmed in other glioma models or in vivo. In another study, Zhu et al. analyzed the data of a total of 2.217 glioma patients in various databases to investigate the role of the galectin gene family in glioma progression [53]. A reliable survival model based on galectin expression was established for glioma patients, and the roles of galectin genes in tumor growth, immunosuppression, preservation of stem cell properties, pro-neuronal-mesenchymal transition, and hypoxia processes were evaluated. According to the findings obtained from this study, the relative expressions of GAL-1, -3, and − 8 were significantly higher in HGGs than in LGGs. In another study by Liu et al., GAL-8 was found to be highly expressed in glioma stem cells, especially in hypoxic regions [54]. High GAL-8 expression in GBM tissues has been associated with poor prognosis in patients, and suppression of GAL-8 expression has been reported to inhibit tumor growth and, thus, prolong survival in GBM mouse models. Consistent with the in vitro and in vivo findings conducted by Metz et al., Zhu et al., and Liu et al., the GAL-8 concentration increased proportionally with grade in the glioma groups in our study. Moreover, GAL-8 showed a 1.890-fold greater risk for diagnosing glioma, a 1.911-fold higher risk also for malignant progression, a 2.167-fold greater risk for developing GBM, and a 1.627-fold higher risk for the presence of glioma in the temporoparietal region. The cross-validation results demonstrated that GAL-8 had a strong discriminatory power between glioma patients and healthy controls with an accuracy rate of 76.7%, and a moderate ability to differentiate between HGG and LGG with an accuracy rate of 68.0%. On the other hand, the performance of GAL-8 in predicting tumor grade, histological tumor subtype, and tumor localization was limited. These findings suggest that GAL-8 may not fully capture the complexity of glioma subtypes, but it still remains a promising candidate for initial noninvasive glioma detection. The area under the ROC curve for serum GAL-8 levels was found to be 0.864, and the risk of glioma was found to be 68.31 times higher at concentrations above the cut-off value. In light of the findings obtained from our study, GAL-8 can be used as a biomarker in the early diagnosis of HGGs and in monitoring disease progress. In addition, the fact that GAL-8 is the most potent marker in malignant gliomas also supports the hypothesis that treatments that suppress this protein can prevent glioma progression.

The interaction of galectins with ITGβ1, a cell surface receptor that mediates physical and functional interactions, plays an important role in tumor progression, apoptosis, angiogenesis, and migration via cell adhesion, cell motility, and intracellular signaling pathways [55, 56]. Therefore, serum ITGβ-1 levels were also evaluated together with galectins in our study and were found to be significantly higher in both tumor groups compared to the healthy group. Additionally, the serum levels of ITGβ-1 in HGGs were greater than those in LGGs, suggesting that ITGβ-1 together with galectins, especially GAL-3 and − 8, can be evaluated as a predictor of glioma severity. ROC curve analysis also supported the idea that ITGβ-1 may be a promising biomarker for the prognosis of gliomas, with the very high AUC value of the ROC curve as 0.905. ITGβ-1 serum value above cut-off was strongly associated with 58.94 times higher risk of glioma development than lower levels. Although there have been several controversial studies on the role of ITGβ-1 in glioma pathology from the perspective of mRNA expression, no study to date has explored ITGβ-1 from the perspective of serum levels. In a study evaluating the expression of ITGβ-1 in normal and neoplastic human brains, an increase in ITGβ1 expression was reported in neoplastic astrocytes in vivo and in vitro, while a similar ITGβ-1 pattern was observed in oligodendroglioma, ependymoma, choroid plexus papilloma, pituitary adenoma, and meningioma cells as in normal cells [57]. On the other hand, GAL-3 and ITGβ-1 were observed to be upregulated in Gli4 cells by Saleh et al. [58]. These findings are consistent with the statistically significant positive correlation between serum GAL-3 and ITGβ-1 obtained in LGGs, in our study. Correlatively with the study by Saleh et al., ITGβ-1 expression has been reported to be increased in resistant GBM cell lines and has been associated with poor prognosis in GBM patients [59]. In another study, ITGβ-1 mRNA and protein levels were analyzed in glioma tumor tissue and peritumoral normal tissue samples around the tumor. In addition, U87 and U251 cell lines suppressing and overexpressing ITGβ-1 expression, respectively, were established, and the role of ITGβ-1 in these cells was evaluated [60]. ITGβ-1 mRNA and protein levels were found to be significantly higher in glial tumors than in normal tissues. Furthermore, ITGβ-1 expression in glial tissues was negatively correlated with patient survival. In the same study, it was also emphasized that the proliferation of U87 cells with suppressed ITGβ-1 expression was significantly reduced, whereas the proliferation of U251 cells overexpressing ITGβ-1 was increased. Sun et al. summarized the upregulation and enhanced invasion of ITGβ-1 in various cancer types, including GBM [61].

HIF-1α is a key regulator of cellular adaptation to hypoxia, modulating critical biological processes such as cell proliferation, apoptosis, angiogenesis, and therapeutic resistance through the regulation of target gene expression [62, 63]. Its role is particularly prominent in HGGs, where it contributes to disease progression and patient survival [64]. In GBMs, HIF-1α is strongly expressed during angiogenesis process [65]. Within necrotic regions, it predominantly localizes in the nuclei of tumor cells, whereas it is primarily found in the cytoplasm in LGGs [64–66]. Furthermore, elevated HIF-1α levels in GBM have been associated with poor survival outcomes [67]. In a study by El-Benhawy et al., serum HIF-1α levels were evaluated in GBM and meningioma patients before and after RT [68]. Compared with healthy volunteers, significant increases in serum HIF-1α were shown before RT in both GBM and meningioma subgroups. After RT, HIF-1α serum levels were significantly enhanced than before RT in GBM patients, whereas they decreased in meningioma patients. Serum HIF-1α levels were significantly higher in GBM patients compared to meningioma patients after RT in this study. In another study to determine the specific features of GBM progression, experimental groups of hypoxias tolerant and susceptible male Wistar rats were established [69]. One month later, the animals were inoculated with GBM cells and sacrificed on day 11 and day 15 after inoculation. Serum HIF-1α levels of both groups were then evaluated, and it was observed that the proliferative activity of GBM cells and HIF-1α levels were increased in the hypoxia-tolerant group on day 15 compared to day 11. HIF-1α has also been shown to be associated with certain aspects of GBM progression, particularly immortalization, invasion, and metastasis. HIF-1α inhibition may be considered a promising therapeutic strategy to prevent cytotoxicity resistance and improve survival by affecting tumor de-differentiation, angiogenesis, and autophagy. In a study conducted by Reszec and colleagues, HIF-1α expression was evaluated in 154 meningioma cases of different grades and 106 glial tumors, including 24 diffuse astrocytomas, 40 anaplastic astrocytomas and 42 GBMs [70]. According to the study results, increased HIF-1α expression was observed in 37.5% of diffuse astrocytoma cases, 27.5% of anaplastic astrocytoma cases, and 83.3% of GBM cases. Furthermore, HIF-1α expression was positive in 55.7% of low-grade meningiomas and 84% of high-grade meningiomas. The findings suggest that HIF-1α expression may be associated with the development and progression of both glial tumors and meningiomas. Similarly, positive levels of HIF-1α expression were observed in 60 cases of GBM [71]. In the group of patients who received only conventional RT after surgery, progression-free survival time significantly differed depending on the level of HIF-1α expression. The findings of the study suggest that high HIF-1α expression may be an indicator of tumor radio-resistance and can be considered as a biomarker to guide the determination of postoperative RT strategies. Supportingly, another study recorded high levels of HIF-1α expression in glioma cells than in normal human astrocytes [72]. It was also reported that HIF-1α expression was greater in hypoxia-treated A172, U87, U251, and Hs683 glioma cells compared to non-hypoxia-treated cells. High HIF-1α expression has been associated with oncogenic functions. In our study, serum HIF-1α levels were significantly higher in the HGG and LGG groups, meaning that HIF-1α may be an indicator of malignancy and may also play a role in glioma development and progression. The AUC of the ROC curve for HIF-1α was 0.686. Moreover, the risk of glioma development was 31.81-fold greater, with HIF-1α serum levels exceeding the cutoff threshold.

As a family of zinc-dependent proteases, MMPs are essential for tissue remodeling and contribute significantly to processes such as wound healing, embryo implantation, tumor invasion, metastatic progression, and angiogenesis [73, 74]. There is considerable evidence in the literature that MMP-2 and − 9 are associated with glioma by increasing cancer cell growth, migration, invasion, and angiogenesis [75–77]. However, the findings regarding the serum levels of MMP-2 and − 9 exhibit inconsistencies. In a study conducted by Sincevičiūtė et al., the mean MMP-2 concentration measured in the serum of glioma patients was lower than control subjects [78]. Similarly, the MMP-2 concentration was significantly lower in grade II gliomas compared to the healthy controls. On the other hand, the increase in the MMP-2 protein concentration in advanced gliomas was interpreted as supporting the role of this protein in malignant progression, and a tendency toward shorter survival was shown with higher MMP-2 protein levels in patient serum. It has been reported that MMP-2 has an important role in glioma pathogenesis and can be used as a potential molecular marker for tumor progression. Conversely, Urbanaviciute et al. did not demonstrate significant differences in protein expression in serum between astrocytoma grades and the control group and reported no relationship between elevated MMP-2 protein expression and patient survival time [79]. Another research group has examined MMP-2 protein expression in astrocytic brain tissue [80]. Findings indicate that MMP-2 expression is significantly elevated in GBM than in normal brain tissue, diffuse astrocytoma, and anaplastic astrocytoma. Furthermore, Ramachandran et al. reported that increased MMP-2 expression was correlated with reduced overall survival in patients diagnosed with grade II–IV astrocytic tumors in the same study. Supportingly, significantly higher serum MMP-2 levels were then shown in GBM patients as compared to the controls [81]. In another study performed by Liu and Li on a total of 220 GBM patients, the preoperative serum levels of MMP-2 and − 9 were evaluated [82]. In recurrent patients, elevated serum levels of MMP-2 and − 9 were observed compared to non-recurrent patients. Serum MMP-2 levels showed a positive correlation with MMP-9 in recurrent patients. Additionally, ROC analyses revealed that both MMP-2 and − 9 effectively distinguished between recurrent and non-recurrent patients, with AUC values of 0.883 and 0.900, respectively. Moreover, in GBM, increased expression of MMP-2 and − 9 is reported to be associated with the malignancy of gliomas [83, 84]. In contrast, a study examining the correlation between serum MMP-9 levels and disease status or survival in glioma patients revealed no statistically significant association between serum MMP-9 levels and radiographic disease status among LGGs, anaplastic gliomas, and GBMs [85]. Among GBM patients, long-term increases in MMP-9 were weakly associated with shorter survival. The authors conclude that serum MMP-9 does not provide any utility in determining glioma disease status and is not a clinically meaningful prognostic marker of survival. In our study, elevated MMP-2 and − 9 levels were obtained in HGG and LGG compared with healthy controls. Additionally, the serum levels of both MMP-2 and − 9 in HGGs were measured as enhanced compared to the LGGs. Our increased serum findings are similar to those of the studies by Ramachandran et al., Shehan et al., Xue et al., and Zhou et al. We also found that MMP-9 effectively distinguished between healthy controls and patients with gliomas, with the AUC of 0.852. The data support the results obtained by Liu and colleagues from the ROC curve analysis for MMP-9. A significant elevation in the risk of glioma, with a 132.48-fold increase, was found at MMP-9 concentrations over the cut-off value. These results highlight the potential of MMP-9 as a diagnostic tool and a marker for tracking the progression of gliomas.

In summary, the combined biomarker panel comprising GAL-8, ITGβ-1, and HIF-1α demonstrated excellent diagnostic performance with an AUC of 0.959, 85% sensitivity and 94% specificity at the optimal threshold, highlighting its potential utility in distinguishing glioma patients from healthy individuals.

This study has several important limitations that warrant consideration. The fifth edition of the WHO Classification of Tumors of the CNS, published in 2021, updated glioma classification by incorporating molecular diagnostics alongside histological and immunohistochemical features [27]. According to the WHO CNS5 criteria, molecular markers such as IDH mutation status, 1p/19q codeletion, ATRX, and TP53 are essential for accurate tumor classification and prognostic stratification of gliomas. However, detailed molecular diagnostic data were not available for all patients in our cohort at the time of data collection. Due to the limited nature of the dataset and limited access to molecular pathology during the diagnostic period, glioma classification in this study was based solely on histopathological criteria in accordance with WHO guidelines. In this context, only immunohistochemical markers GFAP expression and Ki-67 proliferation index were available across cases. obtained from patient data. This limitation may have introduced biological heterogeneity within the study groups and could have influenced the interpretability of our proposed biomarker panel in gliomas with differing genetic profiles. Future prospective studies incorporating comprehensive molecular profiling will be important to validate the clinical utility of GAL-8, ITGβ-1, and HIF-1α in well-characterized glioma subtypes. One another important limitation of this study is the relatively small sample size, which may restrict the generalizability of the results, particularly within specific glioma subtypes. While the statistical associations observed were significant and consistent, validation of our findings in larger, prospective, and multicenter cohorts is warranted to ensure broader applicability. Future studies should also consider including more diverse and demographically heterogeneous populations to strengthen the external validity of the proposed biomarker panel comprising GAL-8, ITGβ-1, and HIF-1α.

In future studies, the spatial co-localization of GAL-8 and HIF-1α at the glioma tissue level should be assessed by immunohistochemistry or multiplex imaging techniques. Such analyses may help to clarify whether these markers interact in hypoxia-induced microdomains such as pseudopalisade necrotic areas and may provide further insight into the mechanistic link between hypoxia, immune modulation, and glioma progression. Determining the spatial relationship between these biomarkers may also help to identify glioma subpopulations with distinct biological behavior, thereby increasing the diagnostic and therapeutic potential of the panel. In particular, evaluation of GAL-8 localization in hypoxia-associated histological regions, such as pseudopalisading necrotic areas, would provide more direct evidence for the role of GAL-8 in the hypoxic microenvironment of gliomas and in the occurrence of hypoxia-induced gliomagenesis. Additionally, given the emerging evidence supporting the immunomodulatory functions of GAL-8, future studies should investigate its association with tumor-infiltrating immune cell subsets particularly CD8⁺ cytotoxic T lymphocytes, regulatory T cells, and myeloid-derived suppressor cells, within the glioma microenvironment. Integrating serum and tissue-level GAL-8 quantification with immune cell profiling through immunohistochemistry/flow cytometry could provide valuable insights into the role of GAL-8 in regulating tumor-induced immunosuppression. Such investigations would not only enhance the mechanistic understanding of glioma progression but also help establish GAL-8 as a potential immunotherapeutic target in HGGs. Another important direction for future research is to examine GAL-8 in the context of glioma stemness and treatment resistance. Future studies integrating stem cell marker profiling with spatial expression analyses will be critical to determine whether GAL-8 contributes to glioma maintenance, resilience, and resistance mechanisms.

## Conclusions

Gliomas are among the most common primary brain tumors and are characterized by their aggressive nature and poor prognosis. Despite advances in surgical techniques, RT, and CT, the treatment of glial tumors remains challenging, primarily due to their invasive growth and high recurrence rates. The difficulty of making early and accurate diagnoses further complicates clinical management. Therefore, identifying reliable biomarkers that reflect tumor progression and response to treatment is essential for improving patient outcomes. In this study, we evaluated the serum levels of GAL-1, -3, and − 8, along with ITGβ-1, HIF-1α, MMP-2, and − 9, as potential diagnostic markers in glioma.

This study is the first in the Turkish population to evaluate serum GAL-1, -3, and − 8 levels together clinically in glioma tumor pathologies with high angiogenic activity, and it sheds light on the role of increased serum galectin levels in the promotion of LGGs to HGG. In this respect, this study may contribute to studies on glioma treatment and gliomagenesis inhibition by bringing a new perspective on the targeting of galectins.

Furthermore, the observed correlations between galectin levels and tumor grade highlight the potential of these biomarkers as valuable indicators for tumor grading and as therapeutic targets. Although no significant differences were found in serum HIF-1α levels between tumor grades, its consistent elevation underscores its relevance in glioma biology. GAL-8, ITGβ-1, and HIF-1α are considered to be usable as a panel of non-invasive biomarkers.

Given the persistent challenge of glioma treatment due to its invasive and recurrent nature, targeting galectin-mediated pathways might offer novel therapeutic avenues. Future studies are warranted to validate these findings in larger cohorts and explore the molecular mechanisms underlying galectin interactions in gliomagenesis. This study provides a crucial step toward understanding the clinical utility of serum galectins in gliomas and contributes to the development of potential biomarker-driven diagnosis strategies.

## Data Availability

No datasets were generated or analysed during the current study.

## Abbreviations

(CNS): Central Nervous System
(GAL-1): Galectin-1
(GAL-3): Galectin-3
(GAL-8): Galectin-8
(ITGβ1): Integrin β1
(ECM): Extracellular matrix
(MMP): Matrix metalloproteinase
(MMP-2): Matrix metalloproteinase-2
(MMP-9): Matrix metalloproteinase-9
(HIF-1α): Hypoxia-inducible factor-1α
(LGGs): Low-grade gliomas
(HGGs): High-grade gliomas
(RT): Radiotherapy
(CT): Chemotherapy
(CV): Coefficient of variation
(One-way ANOVA): One-way analysis of variance
(OR): Odds Ratio
(CI): Confidence Interval
(ROC): Receiver operating characteristic
(AUC): The area under the curve
(GFAP): Glial Fibrillary Acidic Protein
(BMI): Body mass index
(GBM): Glioblastoma Multiforme
(BBB): Blood-brain barrier
(SD): Standard deviation

## References

[1] Jemal A, Siegel R, Ward E, Hao Y, Xu J, Thun MJ (2009) Cancer statistics, 2009. CA Cancer J Clin 59:225–249. 10.3322/caac.20006 19474385 10.3322/caac.20006

[2] Louis DN, Perry A, Reifenberger G, von Deimling A, Figarella-Branger D, Cavenee WK, Ohgaki H, Wiestler OD, Kleihues P, Ellison DW (2016) The 2016 world health organization classification of tumors of the central nervous system: a summary. Acta Neuropathol 131:803–820. 10.1007/s00401-016-1545-1 27157931 10.1007/s00401-016-1545-1

[3] Liu J, Li C, Wang Y, Ji P, Guo S, Zhai Y, Wang N, Lou M, Xu M, Chao M, Jiao Y, Zhao W, Feng F, Qu Y, Ge S, Wang L (2021) Prognostic and predictive factors in elderly patients with glioblastoma: A Single-Center retrospective study. Front Aging Neurosci 13:777962. 10.3389/fnagi.2021.777962 35173600 10.3389/fnagi.2021.777962 PMC8841486

[4] Elola MT, Wolfenstein-Todel C, Troncoso MF, Vasta GR, Rabinovich GA (2007) Galectins: matricellular glycan-binding proteins linking cell adhesion, migration, and survival. Cell Mol Life Sci 64:1679–1700. 10.1007/s00018-007-7044-8 17497244 10.1007/s00018-007-7044-8 PMC11136145

[5] Rhodes DH, Pini M, Castellanos KJ, Montero-Melendez T, Cooper D, Perretti M, Fantuzzi G (2013) Adipose tissue-specific modulation of galectin expression in lean and obese mice: evidence for regulatory function. Obes (Silver Spring) 21:310–319. 10.1002/oby.20016 10.1002/oby.20016 PMC3610793 23401338

[6] Sturm A, Lensch M, Andre S, Kaltner H, Wiedenmann B, Rosewicz S, Dignass AU, Gabius HJ (2004) Human galectin-2: novel inducer of T cell apoptosis with distinct profile of caspase activation. J Immunol 173:3825–3837. 10.4049/jimmunol.173.6.3825 15356130 10.4049/jimmunol.173.6.3825

[7] Xue H, Liu L, Zhao Z, Zhang Z, Guan Y, Cheng H, Zhou Y, Tai G (2017) The N-terminal tail coordinates with carbohydrate recognition domain to mediate galectin-3 induced apoptosis in T cells. Oncotarget 8:49824–49838. 10.18632/oncotarget.17760 28548942 10.18632/oncotarget.17760 PMC5564810

[8] Jensen RL (2006) Hypoxia in the tumorigenesis of gliomas and as a potential target for therapeutic measures. Neurosurg Focus 20:E24. 10.3171/foc.2006.20.4.16 16709030 10.3171/foc.2006.20.4.16

[9] D’Abaco GM, Kaye AH (2007) Integrins: molecular determinants of glioma invasion. J Clin Neurosci 14:1041–1048. 10.1016/j.jocn.2007.06.019 17954373 10.1016/j.jocn.2007.06.019

[10] Hynes RO (2002) Integrins: bidirectional, allosteric signaling machines. Cell 110:673–687. 10.1016/s0092-8674(02)00971-6 12297042 10.1016/s0092-8674(02)00971-6

[11] Decaestecker C, Debeir O, Van Ham P, Kiss R (2007) Can anti-migratory drugs be screened in vitro? A review of 2D and 3D assays for the quantitative analysis of cell migration. Med Res Rev 27:149–176. 10.1002/med.20078 16888756 10.1002/med.20078

[12] Stillman BN, Mischel PS, Baum LG (2005) New roles for galectins in brain tumors–from prognostic markers to therapeutic targets. Brain Pathol 15:124–132. 10.1111/j.1750-3639.2005.tb00507.x 15912884 10.1111/j.1750-3639.2005.tb00507.x PMC8095905

[13] Camby I, Belot N, Rorive S, Lefranc F, Maurage CA, Lahm H, Kaltner H, Hadari Y, Ruchoux MM, Brotchi J, Zick Y, Salmon I, Gabius HJ, Kiss R (2001) Galectins are differentially expressed in supratentorial pilocytic Astrocytomas, Astrocytomas, anaplastic Astrocytomas and glioblastomas, and significantly modulate tumor astrocyte migration. Brain Pathol 11:12–26. 10.1111/j.1750-3639.2001.tb00377.x 11145198 10.1111/j.1750-3639.2001.tb00377.x PMC8098336

[14] Woessner JF Jr (1999) Matrix metalloproteinase inhibition. From the jurassic to the third millennium. Ann N Y Acad Sci 878:388–403. 10.1111/j.1749-6632.1999.tb07697.x 10415743 10.1111/j.1749-6632.1999.tb07697.x

[15] Yu AE, Hewitt RE, Kleiner DE, Stetler-Stevenson WG (1996) Molecular regulation of cellular invasion–role of gelatinase A and TIMP-2. Biochem Cell Biol 74:823–831. 10.1139/o96-088 9164651 10.1139/o96-088

[16] Louis DN (2006) Molecular pathology of malignant gliomas. Annu Rev Pathol 1:97–117. 10.1146/annurev.pathol.1.110304.100043 18039109 10.1146/annurev.pathol.1.110304.100043

[17] Rong Y, Durden DL, Van Meir EG, Brat DJ (2006) Pseudopalisading’ necrosis in glioblastoma: a familiar morphologic feature that links vascular pathology, hypoxia, and angiogenesis. J Neuropathol Exp Neurol 65:529–539. 10.1097/00005072-200606000-00001 16783163 10.1097/00005072-200606000-00001

[18] Kaur B, Khwaja FW, Severson EA, Matheny SL, Brat DJ, Van Meir EG (2005) Hypoxia and the hypoxia-inducible-factor pathway in glioma growth and angiogenesis. Neuro Oncol 7:134–153. 10.1215/S1152851704001115 15831232 10.1215/S1152851704001115 PMC1871894

[19] Gagner JP, Law M, Fischer I, Newcomb EW, Zagzag D (2005) Angiogenesis in gliomas: imaging and experimental therapeutics. Brain Pathol 15:342–363. 10.1111/j.1750-3639.2005.tb00119.x 16389946 10.1111/j.1750-3639.2005.tb00119.x PMC8095871

[20] Wei R, Zhou J, Bui B, Liu X (2024) Glioma actively orchestrate a self-advantageous extracellular matrix to promote recurrence and progression. BMC Cancer 24:974. 10.1186/s12885-024-12751-3 39118096 10.1186/s12885-024-12751-3 PMC11308147

[21] Grochans S, Cybulska AM, Siminska D, Korbecki J, Kojder K, Chlubek D, Baranowska-Bosiacka I (2022) Epidemiology of glioblastoma Multiforme-Literature review. Cancers (Basel) 14. 10.3390/cancers14102412 10.3390/cancers14102412 PMC9139611 35626018

[22] Metz C, Doger R, Riquelme E, Cortes P, Holmes C, Shaughnessy R, Oyanadel C, Grabowski C, Gonzalez A, Soza A (2016) Galectin-8 promotes migration and proliferation and prevents apoptosis in U87 glioblastoma cells. Biol Res 49:33. 10.1186/s40659-016-0091-6 27459991 10.1186/s40659-016-0091-6 PMC4962418

[23] Lopes Abath Neto O, Aldape K (2021) Morphologic and molecular aspects of glioblastomas. Neurosurg Clin N Am 32:149–158. 10.1016/j.nec.2021.01.001 33781498 10.1016/j.nec.2021.01.001

[24] Collins VP (2004) Brain tumours: classification and genes. J Neurol Neurosurg Psychiatry 75(Suppl 2):ii2–11. 10.1136/jnnp.2004.040337 15146033 10.1136/jnnp.2004.040337 PMC1765661

[25] Buckner JC (2003) Factors influencing survival in high-grade gliomas. Semin Oncol 30:10–14. 10.1053/j.seminoncol.2003.11.031 14765378 10.1053/j.seminoncol.2003.11.031

[26] DeAngelis LM (2001) Brain tumors. N Engl J Med 344:114–123. 10.1056/NEJM200101113440207 11150363 10.1056/NEJM200101113440207

[27] Louis DN, Perry A, Wesseling P, Brat DJ, Cree IA, Figarella-Branger D, Hawkins C, Ng HK, Pfister SM, Reifenberger G, Soffietti R, von Deimling A, Ellison DW (2021) The 2021 WHO classification of tumors of the central nervous system: a summary. Neuro Oncol 23:1231–1251. 10.1093/neuonc/noab106 34185076 10.1093/neuonc/noab106 PMC8328013

[28] Rubinstein N, Alvarez M, Zwirner NW, Toscano MA, Ilarregui JM, Bravo A, Mordoh J, Fainboim L, Podhajcer OL, Rabinovich GA (2004) Targeted Inhibition of galectin-1 gene expression in tumor cells results in heightened T cell-mediated rejection; A potential mechanism of tumor-immune privilege. Cancer Cell 5:241–251. 10.1016/s1535-6108(04)00024-8 15050916 10.1016/s1535-6108(04)00024-8

[29] Verschuere T, Van Woensel M, Fieuws S, Lefranc F, Mathieu V, Kiss R, Van Gool SW, De Vleeschouwer S (2013) Altered galectin-1 serum levels in patients diagnosed with high-grade glioma. J Neurooncol 115:9–17. 10.1007/s11060-013-1201-8 23824536 10.1007/s11060-013-1201-8

[30] Yamaoka K, Mishima K, Nagashima Y, Asai A, Sanai Y, Kirino T (2000) Expression of galectin-1 mRNA correlates with the malignant potential of human gliomas and expression of antisense galectin-1 inhibits the growth of 9 glioma cells. J Neurosci Res 59:722–730. 10.1002/(SICI)1097-4547(20000315)59:6-722::AID-JNR4-3.0.CO;2-H 10700009 10.1002/(SICI)1097-4547(20000315)59:6<722::AID-JNR4>3.0.CO;2-H

[31] Toussaint LG 3rd, Nilson AE, Goble JM, Ballman KV, James CD, Lefranc F, Kiss R, Uhm JH (2012) Galectin-1, a gene preferentially expressed at the tumor margin, promotes glioblastoma cell invasion. Mol Cancer 11:32. 10.1186/1476-4598-11-32 10.1186/1476-4598-11-32 PMC3407025 22583806

[32] Chou SY, Yen SL, Huang CC, Huang EY (2018) Galectin-1 is a poor prognostic factor in patients with glioblastoma multiforme after radiotherapy. BMC Cancer 18:105. 10.1186/s12885-018-4025-2 29378529 10.1186/s12885-018-4025-2 PMC5789739

[33] Le Mercier M, Lefranc F, Mijatovic T, Debeir O, Haibe-Kains B, Bontempi G, Decaestecker C, Kiss R, Mathieu V (2008) Evidence of galectin-1 involvement in glioma chemoresistance. Toxicol Appl Pharmacol 229:172–183. 10.1016/j.taap.2008.01.009 18313712 10.1016/j.taap.2008.01.009

[34] Dumic J, Dabelic S, Flogel M (2006) Galectin-3: an open-ended story. Biochim Biophys Acta 1760:616–635. 10.1016/j.bbagen.2005.12.020 16478649 10.1016/j.bbagen.2005.12.020

[35] Nangia-Makker P, Hogan V, Raz A (2018) Galectin-3 and cancer stemness. Glycobiology 28:172–181. 10.1093/glycob/cwy001 29315388 10.1093/glycob/cwy001 PMC6279147

[36] An Y, Xu S, Liu Y, Xu X, Philips CA, Chen J, Mendez-Sanchez N, Guo X, Qi X (2021) Role of galectins in the liver diseases: A systematic review and Meta-Analysis. Front Med (Lausanne) 8:744518. 10.3389/fmed.2021.744518 34778306 10.3389/fmed.2021.744518 PMC8578830

[37] Chamseddine S, Yavuz BG, Mohamed YI, Lee SS, Yao JC, Hu ZI, LaPelusa M, Xiao L, Sun R, Morris JS, Hatia RI, Hassan M, Duda DG, Diab M, Mohamed A, Nassar A, Amin HM, Kaseb AO (2024) Circulating Galectin-3: A prognostic biomarker in hepatocellular carcinoma. J Immunother Precis Oncol 7:255–262. 10.36401/JIPO-24-6 39524465 10.36401/JIPO-24-6 PMC11541930

[38] Cheng D, Liang B, Li Y (2015) Serum galectin-3 as a potential marker for gastric cancer. Med Sci Monit 21:755–760. 10.12659/MSM.892386 25765552 10.12659/MSM.892386 PMC4370354

[39] Shimura T, Shibata M, Gonda K, Nakajima T, Chida S, Noda M, Suzuki S, Nakamura I, Ohki S, Takenoshita S (2016) Association between Circulating galectin-3 levels and the immunological, inflammatory and nutritional parameters in patients with colorectal cancer. Biomed Rep 5:203–207. 10.3892/br.2016.696 27446542 10.3892/br.2016.696 PMC4950301

[40] Yi N, Zhao X, Ji J, Xu M, Jiao Y, Qian T, Zhu S, Jiang F, Chen J, Xiao M (2020) Serum galectin-3 as a biomarker for screening, early diagnosis, prognosis and therapeutic effect evaluation of pancreatic cancer. J Cell Mol Med 24:11583–11591. 10.1111/jcmm.15775 32886424 10.1111/jcmm.15775 PMC7576229

[41] Wang H, Song X, Huang Q, Xu T, Yun D, Wang Y, Hu L, Yan Y, Chen H, Lu D, Chen J (2019) LGALS3 promotes treatment resistance in glioblastoma and is associated with tumor risk and prognosis. Cancer Epidemiol Biomarkers Prev 28:760–769. 10.1158/1055-9965.EPI-18-0638 30341098 10.1158/1055-9965.EPI-18-0638

[42] Hu WM, Yang YZ, Zhang TZ, Qin CF, Li XN (2020) LGALS3 is a poor prognostic factor in diffusely infiltrating gliomas and is closely correlated with CD163 + Tumor-Associated macrophages. Front Med (Lausanne) 7:182. 10.3389/fmed.2020.00182 32528967 10.3389/fmed.2020.00182 PMC7254797

[43] He X, Zhang S, Chen J, Li D (2019) Increased LGALS3 expression independently predicts shorter overall survival in patients with the proneural subtype of glioblastoma. Cancer Med 8:2031–2040. 10.1002/cam4.2075 30848102 10.1002/cam4.2075 PMC6536958

[44] Ikemori RY, Machado CM, Furuzawa KM, Nonogaki S, Osinaga E, Umezawa K, de Carvalho MA, Verinaud L, Chammas R (2014) Galectin-3 up-regulation in hypoxic and nutrient deprived microenvironments promotes cell survival. PLoS ONE 9:e111592. 10.1371/journal.pone.0111592 25369297 10.1371/journal.pone.0111592 PMC4219723

[45] Ajarrag S, St-Pierre Y (2021) Galectins in glioma: current roles in Cancer progression and future directions for improving treatment. Cancers (Basel) 13. 10.3390/cancers13215533 10.3390/cancers13215533 PMC8582867 34771696

[46] Barrow H, Guo X, Wandall HH, Pedersen JW, Fu B, Zhao Q, Chen C, Rhodes JM, Yu LG (2011) Serum galectin-2, -4, and– 8 are greatly increased in colon and breast cancer patients and promote cancer cell adhesion to blood vascular endothelium. Clin Cancer Res 17:7035–7046. 10.1158/1078-0432.CCR-11-1462 21933892 10.1158/1078-0432.CCR-11-1462

[47] Beyer S, Wehrmann M, Meister S, Kolben TM, Trillsch F, Burges A, Czogalla B, Schmoeckel E, Mahner S, Jeschke U, Kolben T (2022) Galectin-8 and– 9 as prognostic factors for cervical cancer. Arch Gynecol Obstet 306:1211–1220. 10.1007/s00404-022-06449-9 35377045 10.1007/s00404-022-06449-9 PMC9470666

[48] Chakraborty A, Perez M, Carroll JD, Antonopoulos A, Dell A, Ortega L, Mohammed NBB, Wells M, Staudinger C, Griswold A, Chandler KB, Marrero C, Jimenez R, Tani Y, Wilmott JS, Thompson JF, Wang W, Sackstein R, Scolyer RA, Murphy GF, Haslam SM, Dimitroff CJ (2023) Hypoxia Controls the Glycome Signature and Galectin-8-Ligand Axis to Promote Protumorigenic Properties of Metastatic Melanoma. J Invest Dermatol 143: 456–469 e458 10.1016/j.jid.2022.07.033 10.1016/j.jid.2022.07.033 PMC10123958 36174713

[49] Ferragut F, Cagnoni AJ, Colombo LL, Sanchez Terrero C, Wolfenstein-Todel C, Troncoso MF, Vanzulli SI, Rabinovich GA, Marino KV, Elola MT (2019) Dual knockdown of Galectin-8 and its glycosylated ligand, the activated leukocyte cell adhesion molecule (ALCAM/CD166), synergistically delays in vivo breast cancer growth. Biochim Biophys Acta Mol Cell Res 1866:1338–1352. 10.1016/j.bbamcr.2019.03.010 30905597 10.1016/j.bbamcr.2019.03.010

[50] Gentilini LD, Jaworski FM, Tiraboschi C, Perez IG, Kotler ML, Chauchereau A, Laderach DJ, Compagno D (2017) Stable and high expression of Galectin-8 tightly controls metastatic progression of prostate cancer. Oncotarget 8:44654–44668. 10.18632/oncotarget.17963 28591719 10.18632/oncotarget.17963 PMC5546508

[51] Labrie M, De Araujo LOF, Communal L, Mes-Masson AM, St-Pierre Y (2017) Tissue and plasma levels of galectins in patients with high grade serous ovarian carcinoma as new predictive biomarkers. Sci Rep 7:13244. 10.1038/s41598-017-13802-5 29038585 10.1038/s41598-017-13802-5 PMC5643335

[52] Nagy N, Bronckart Y, Camby I, Legendre H, Lahm H, Kaltner H, Hadari Y, Van Ham P, Yeaton P, Pector JC, Zick Y, Salmon I, Danguy A, Kiss R, Gabius HJ (2002) Galectin-8 expression decreases in cancer compared with normal and dysplastic human colon tissue and acts significantly on human colon cancer cell migration as a suppressor. Gut 50:392–401. 10.1136/gut.50.3.392 11839721 10.1136/gut.50.3.392 PMC1773143

[53] Zhu H, Liu D, Cheng L, Liu J, Wang G, Li H, Zhang Y, Mi H, Zhang S, Shu K, Yu X (2022) Prognostic value and biological function of galectins in malignant glioma. Front Oncol 12:834307. 10.3389/fonc.2022.834307 35814469 10.3389/fonc.2022.834307 PMC9263596

[54] Liu D, Zhu H, Cheng L, Li R, Ma X, Wang J, Wang J, Zhang S, Li Y, Shu K, Yu X, Li C (2024) Hypoxia-induced galectin-8 maintains stemness in glioma stem cells via autophagy regulation. Neuro Oncol 26:872–888. 10.1093/neuonc/noad264 38158714 10.1093/neuonc/noad264 PMC11066898

[55] Bartik P, Maglott A, Entlicher G, Vestweber D, Takeda K, Martin S, Dontenwill M (2008) Detection of a hypersialylated beta1 integrin endogenously expressed in the human Astrocytoma cell line A172. Int J Oncol 32:1021–1031 18425328

[56] Hamidi H, Ivaska J (2018) Every step of the way: integrins in cancer progression and metastasis. Nat Rev Cancer 18:533–548. 10.1038/s41568-018-0038-z 30002479 10.1038/s41568-018-0038-z PMC6629548

[57] Paulus W, Baur I, Schuppan D, Roggendorf W (1993) Characterization of integrin receptors in normal and neoplastic human brain. Am J Pathol 143:154–163 8317546 PMC1886949

[58] Saleh A, Marhuenda E, Fabre C, Hassani Z, Weille J, Boukhaddaoui H, Guelfi S, Maldonado IL, Hugnot JP, Duffau H, Bauchet L, Cornu D, Bakalara N (2019) A novel 3D nanofibre scaffold conserves the plasticity of glioblastoma stem cell invasion by regulating galectin-3 and integrin-beta1 expression. Sci Rep 9:14612. 10.1038/s41598-019-51108-w 31601895 10.1038/s41598-019-51108-w PMC6787018

[59] Petras M, Lajtos T, Friedlander E, Klekner A, Pintye E, Feuerstein BG, Szollosi J, Vereb G (2013) Molecular interactions of ErbB1 (EGFR) and integrin-beta1 in Astrocytoma frozen sections predict clinical outcome and correlate with Akt-mediated in vitro radioresistance. Neuro Oncol 15:1027–1040. 10.1093/neuonc/not046 23595626 10.1093/neuonc/not046 PMC3714153

[60] Min W, Zou C, Dai D, Zuo Q, Chen C, Xu J, Li Y, Yue Z (2020) Integrin Beta 1 Promotes Glioma Cell Proliferation by Negatively Regulating the Notch Pathway. J Oncol 2020: 8297017 10.1155/2020/8297017 10.1155/2020/8297017 PMC7512099 33014056

[61] Sun L, Guo S, Xie Y, Yao Y (2023) The characteristics and the multiple functions of integrin beta1 in human cancers. J Transl Med 21:787. 10.1186/s12967-023-04696-1 37932738 10.1186/s12967-023-04696-1 PMC10629185

[62] Liu ZJ, Semenza GL, Zhang HF (2015) Hypoxia-inducible factor 1 and breast cancer metastasis. J Zhejiang Univ Sci B 16:32–43. 10.1631/jzus.B1400221 25559953 10.1631/jzus.B1400221 PMC4288942

[63] Zhao Y, Xing C, Deng Y, Ye C, Peng H (2024) HIF-1alpha signaling: essential roles in tumorigenesis and implications in targeted therapies. Genes Dis 11:234–251. 10.1016/j.gendis.2023.02.039 37588219 10.1016/j.gendis.2023.02.039 PMC10425810

[64] Wang G, Wang JJ, Fu XL, Guang R, To ST (2017) Advances in the targeting of HIF-1alpha and future therapeutic strategies for glioblastoma multiforme (Review). Oncol Rep 37:657–670. 10.3892/or.2016.5309 27959421 10.3892/or.2016.5309

[65] Zagzag D, Zhong H, Scalzitti JM, Laughner E, Simons JW, Semenza GL (2000) Expression of hypoxia-inducible factor 1alpha in brain tumors: association with angiogenesis, invasion, and progression. Cancer 88:2606–2618 10861440

[66] Sharpe MA, Baskin DS (2016) Monoamine oxidase B levels are highly expressed in human gliomas and are correlated with the expression of HiF-1alpha and with transcription factors Sp1 and Sp3. Oncotarget 7:3379–3393. 10.18632/oncotarget.6582 26689994 10.18632/oncotarget.6582 PMC4823113

[67] Sfifou F, Hakkou EM, Bouaiti EA, Slaoui M, Errihani H, Al Bouzidi A, Abouqal R, El Ouahabi A, Cherradi N (2021) Correlation of immunohistochemical expression of HIF-1alpha and IDH1 with clinicopathological and therapeutic data of Moroccan glioblastoma and survival analysis. Ann Med Surg (Lond) 69:102731. 10.1016/j.amsu.2021.102731 34466221 10.1016/j.amsu.2021.102731 PMC8384773

[68] El-Benhawy SA, Sakr OA, Fahmy EI, Ali RA, Hussein MS, Nassar EM, Salem SM, Abu-Samra N, Elzawawy S (2022) Assessment of serum hypoxia biomarkers Pre- and Post-radiotherapy in patients with brain tumors. J Mol Neurosci 72:2303–2312. 10.1007/s12031-022-02065-z 36121548 10.1007/s12031-022-02065-z PMC9726784

[69] Dzhalilova DS, Zolotova NA, Mkhitarov VA, Kosyreva AM, Tsvetkov IS, Khalansky AS, Alekseeva AI, Fatkhudinov TH, Makarova OV (2023) Morphological and molecular-biological features of glioblastoma progression in tolerant and susceptible to hypoxia Wistar rats. Sci Rep 13:12694. 10.1038/s41598-023-39914-9 37542119 10.1038/s41598-023-39914-9 PMC10403616

[70] Reszec J, Rutkowski R, Chyczewski L (2013) The expression of hypoxia-inducible factor-1 in primary brain tumors. Int J Neurosci 123:657–662. 10.3109/00207454.2013.789874 23550771 10.3109/00207454.2013.789874

[71] Irie N, Matsuo T, Nagata I (2004) Protocol of radiotherapy for glioblastoma according to the expression of HIF-1. Brain Tumor Pathol 21:1–6. 10.1007/BF02482169 15696961 10.1007/BF02482169

[72] Lin YH, Wu ZY (2023) Hypoxia-inducible factor 1α (HIF-1α)-activated Gli1 induces invasion and EMT by H3K4 methylation in glioma cells. Oncologie 25:71–79. 10.1515/oncologie-2023-0004

[73] Ray JM, Stetler-Stevenson WG (1994) The role of matrix metalloproteases and their inhibitors in tumour invasion, metastasis and angiogenesis. Eur Respir J 7:2062–2072 7533104

[74] Rosenberg GA (1995) Matrix metalloproteinases in brain injury. J Neurotrauma 12:833–842. 10.1089/neu.1995.12.833 8594211 10.1089/neu.1995.12.833

[75] Forsyth PA, Wong H, Laing TD, Rewcastle NB, Morris DG, Muzik H, Leco KJ, Johnston RN, Brasher PM, Sutherland G, Edwards DR (1999) Gelatinase-A (MMP-2), gelatinase-B (MMP-9) and membrane type matrix metalloproteinase-1 (MT1-MMP) are involved in different aspects of the pathophysiology of malignant gliomas. Br J Cancer 79:1828–1835. 10.1038/sj.bjc.6690291 10206300 10.1038/sj.bjc.6990291 PMC2362801

[76] Guo P, Imanishi Y, Cackowski FC, Jarzynka MJ, Tao HQ, Nishikawa R, Hirose T, Hu B, Cheng SY (2005) Up-regulation of angiopoietin-2, matrix metalloprotease-2, membrane type 1 metalloprotease, and laminin 5 gamma 2 correlates with the invasiveness of human glioma. Am J Pathol 166:877–890. 10.1016/s0002-9440(10)62308-5 15743799 10.1016/s0002-9440(10)62308-5 PMC1602359

[77] Sawaya RE, Yamamoto M, Gokaslan ZL, Wang SW, Mohanam S, Fuller GN, McCutcheon IE, Stetler-Stevenson WG, Nicolson GL, Rao JS (1996) Expression and localization of 72 kda type IV collagenase (MMP-2) in human malignant gliomas in vivo. Clin Exp Metastasis 14:35–42. 10.1007/BF00157684 8521615 10.1007/BF00157684

[78] Sinceviciute R, Vaitkiene P, Urbanaviciute R, Steponaitis G, Tamasauskas A, Skiriute D (2018) MMP2 is associated with glioma malignancy and patient outcome. Int J Clin Exp Pathol 11:3010–3018 31938426 PMC6958083

[79] Urbanaviciute R, Skauminas K, Skiriute D (2020) The Evaluation of AREG, MMP-2, CHI3L1, GFAP, and OPN Serum Combined Value in Astrocytic Glioma Patients’ Diagnosis and Prognosis. Brain Sci 10. 10.3390/brainsci10110872 10.3390/brainsci10110872 PMC7699177 33227903

[80] Ramachandran RK, Sorensen MD, Aaberg-Jessen C, Hermansen SK, Kristensen BW (2017) Expression and prognostic impact of matrix metalloproteinase-2 (MMP-2) in Astrocytomas. PLoS ONE 12:e0172234. 10.1371/journal.pone.0172234 28234925 10.1371/journal.pone.0172234 PMC5325257

[81] Shehan JG, Vengoji S, Rachagani R, Zhang S, Mallya Y, Pai K, Kaur P, Jain S, Kueh M, White A, Aizenberg M, McComb M, Smith RD, Cohen L, Batra MZ, S.K.; and, Shonka N (2019) MMP-2 AND NGAL AS BIOMARKERS IN GLIOBLASTOMA: A PILOT STUDY. Med Res Archives 7:1–17

[82] Liu W, Li Z (2023) Diagnostic performance of perfusion-weighted imaging combined with serum MMP-2 and– 9 levels in tumor recurrence after postoperative concomitant chemoradiotherapy of glioblastoma. J Clin Ultrasound 51:563–570. 10.1002/jcu.23402 36435971 10.1002/jcu.23402

[83] Xue Q, Cao L, Chen XY, Zhao J, Gao L, Li SZ, Fei Z (2017) High expression of MMP9 in glioma affects cell proliferation and is associated with patient survival rates. Oncol Lett 13:1325–1330. 10.3892/ol.2017.5567 28454256 10.3892/ol.2017.5567 PMC5403257

[84] Zhou W, Yu X, Sun S, Zhang X, Yang W, Zhang J, Zhang X, Jiang Z (2019) Increased expression of MMP-2 and MMP-9 indicates poor prognosis in glioma recurrence. Biomed Pharmacother 118:109369. 10.1016/j.biopha.2019.109369 31545229 10.1016/j.biopha.2019.109369

[85] Iwamoto FM, Hottinger AF, Karimi S, Riedel E, Dantis J, Jahdi M, Panageas KS, Lassman AB, Abrey LE, Fleisher M, Deangelis LM, Holland EC, Hormigo A (2011) Longitudinal prospective study of matrix metalloproteinase-9 as a serum marker in gliomas. J Neurooncol 105:607–612. 10.1007/s11060-011-0628-z 21710351 10.1007/s11060-011-0628-z PMC7398421

